# Global trends and risk factors in gastric cancer: a comprehensive analysis of the Global Burden of Disease Study 2021 and multi-omics data

**DOI:** 10.7150/ijms.104437

**Published:** 2025-01-01

**Authors:** Liqun Zhang, Qian Dong, Yuanhe Wang, Xiaoxi Li, Chunning Li, Fang Li, Jingdong Zhang

**Affiliations:** 1 Medical Oncology Department of Gastrointestinal Cancer, Cancer Hospital of Dalian University of Technology, Liaoning Cancer Hospital & Institute, No.44 Xiaoheyan Road, Dadong District, Shenyang 110042, Liaoning Province, China.; 2 School of Biomedical Engineering, Faculty of Medicine, Dalian University of Technology, No.2 Linggong Road, Ganjingzi District, Dalian 116024, Liaoning Province, China.; 3 Central Laboratory, Cancer Hospital of Dalian University of Technology, Liaoning Cancer Hospital & Institute, No.44 Xiaoheyan Road, Dadong District, Shenyang 110042, Liaoning Province, China.; 4 Department of Hepatobiliary Surgery, Cancer Hospital of Dalian University of Technology, Liaoning Cancer Hospital & Institute, No.44 Xiaoheyan Road, Dadong District, Shenyang 110042, Liaoning Province, China.

**Keywords:** Gastric cancer, Global burden of disease, Gut microbiome, Risk factors, Biomarkers

## Abstract

**Background:** Gastric cancer (GC) remains a significant global health challenge. This study aimed to comprehensively analyze GC epidemiology and risk factors to inform prevention and intervention strategies.

**Methods:** We analyzed the Global Burden of Disease Study 2021 data, conducted 16 different machine learning (ML) models of NHANES data, performed Mendelian randomization (MR) studies on disease phenotypes, dietary preferences, microbiome, blood-based markers, and integrated differential gene expression and expression quantitative trait loci (eQTL) data from multiple cohorts to identify factors associated with GC risk.

**Results:** Global age-standardized disability-adjusted life year rates (ASDR) for GC declined from 886.24 to 358.42 per 100,000 population between 1990 and 2030, with significant regional disparities. Despite this decline, total disability-adjusted life years show a concerning upward trend from 2015, rising from approximately 22.9 million to a projected 24.3 million by 2030. The slope index of inequality shifted from 87 in 1990 to -184 in 2021, indicating a reversal in GC burden distribution, with higher ASDR now associated with lower socio-demographic index countries. The ML models analysis identified higher levels of clinical characteristics such as phosphorus, calcium, eosinophils percent, and triglycerides, as well as lower levels of iron and monocyte percent, may be associated with an increased risk of GC. MR analyses revealed causal associations between GC risk and disease phenotypes such as *Helicobacter pylori* infection, chronic gastritis, obesity, depression, and dietary preferences such as dairy and processed meats. Gut microbiome analysis showed associations with microbiome such as *Phascolarctobacterium* and *Ruminococcaceae* species. Blood-based markers analysis identified protective and risk effects for cortisol, glutamate, nicotinamide, Natural Killer %lymphocyte, CD4-CD8- T cell Absolute Count, Phosphatidylcholine (16:0_18:1), and Interleukin-1-alpha. Integrated genomic analysis identified 10 genes significantly associated with GC risk, with strong evidence for colocalization in genes such as *CCR6* and *PILRB*.

**Conclusions:** This systematic analysis reveals complex global trends in GC burden and identifies novel clinical, disease phenotypes, dietary preferences, microbial, blood-based, and genetic risk factors. These findings provide potential targets for improved risk stratification, prevention, and intervention strategies to reduce the global burden of GC.

## Introduction

Gastric cancer (GC) remains a significant global health challenge, ranking as the fifth most common cancer and the fourth leading cause of cancer-related deaths worldwide 1. Its high mortality rate and substantial socioeconomic impact underscore the critical need for exhaustive research into its epidemiology, risk factors, and underlying biological mechanisms. Understanding the multifaceted nature of GC is crucial for developing effective prevention strategies, improving early detection, and enhancing treatment outcomes.

The Global Burden of Disease (GBD) study has provided valuable insights into the changing landscape of GC epidemiology 2. Previous analyses based on GBD data from 1990 to 2019 have illuminated trends in GC epidemiology 3. However, the rapidly evolving nature of cancer epidemiology necessitates continuous updates and more current analyses. Our study extends this temporal framework to 2021, incorporating the most recent data available, to provide a more contemporary and relevant picture of GC burden worldwide 4. While the GBD database offers a wealth of information on disease burden, its utility in elucidating the complex risk factor profile of GC is limited. The multifactorial etiology of GC, involving an intricate interplay of genetic, environmental, and lifestyle factors, demands a more extensive approach to risk factor analysis 5-7. To address this gap, our study integrates a wide array of potential risk factors and biomarkers, leveraging advanced statistical and bioinformatic techniques to provide a holistic view of GC risk.

In addition to analyzing the burden trend of GC by GBD 2021, this study also evaluates the relationships between GC risk and a wide range of factors, including clinical characteristics, comorbidities, food preferences, gut microbiota, blood metabolites, immune cells, lipids, inflammatory markers, and genetic markers: Utilizing data from the National Health and Nutrition Examination Survey (NHANES), we apply 16 different machine learning models to identify clinical parameters associated with GC risk. This approach allows for the discovery of novel clinical biomarkers and risk factors. Through Mendelian randomization (MR) analyses, we examine the causal relationships between various disease phenotypes, dietary preferences, and GC risk. This method, using data from the FinnGen R10, UK Biobank-SAIGE GWAS, *Helicobacter pylori* infection, and food-liking databases, provides robust evidence for causal associations while minimizing confounding factors 8-11. We also investigate the associations between GC risk and an in-depth panel of blood-based markers, including metabolites, immune cell populations, lipid species, and inflammatory proteins 12-15. Recognizing the emerging role of the gut microbiome in cancer development, we analyze data from Finnish and German cohorts to explore associations between specific microbial taxa and GC risk 16, 17. At last, we conduct an integrated analysis of differential gene expression, expression quantitative trait loci (eQTL) data 18. This approach, combining data from The Cancer Genome Atlas (TCGA) and Genotype-Tissue Expression (GTEx) databases with summary-data-based Mendelian randomization (SMR) analysis, provides a systematic view of the genetic and epigenetic landscape of GC risk 19-22. By synthesizing these diverse data sources and analytical approaches, our study aims to provide a nuanced and multidimensional understanding of GC risk. This detailed approach not only enhances our knowledge of GC etiology but also has the potential to inform more effective strategies for prevention, early detection, and personalized treatment.

## Methods

**Figure 1:**
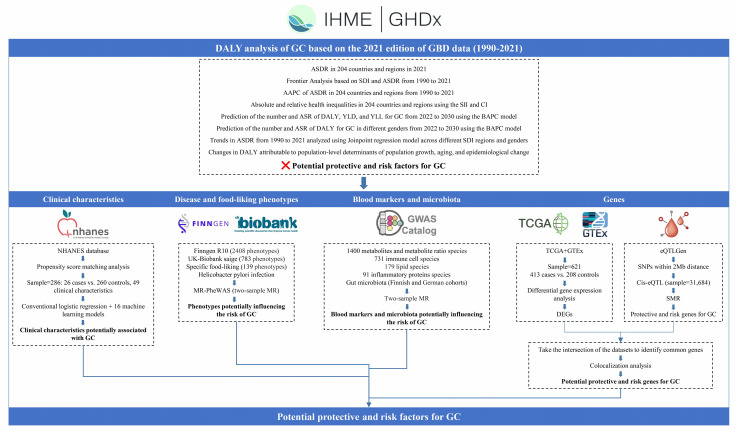
Schematic overview of the study design and methodologies.

Figure 1 delineates the extensive, multi-stage research design employed in this investigation of GC epidemiology and associated risk factors. The study commenced with an extensive analysis of GC epidemiology utilizing the GBD database. This analysis encompassed the examination of age-standardized disability-adjusted life year rates (ASDR) across 204 countries and territories, frontier analysis based on the socio-demographic index (SDI), and the projection of future trends in disability-adjusted life years (DALYs) employing Bayesian Age-Period-Cohort (BAPC) models. Health inequalities were assessed using the slope index of inequality (SII) and concentration index.

Subsequently, the NHANES database was utilized to analyze clinical characteristics associated with GC. Following data cleaning of the NHANES database, we employed propensity score matching (PSM) to extract samples. Subsequently, we conducted analyses using both conventional logistic regression and an array of 16 machine learning models.

Disease and food-liking phenotypes association studies were conducted using the FinnGen R10, UK Biobank SAIGE, *Helicobacter pylori* infection (anti-*Helicobacter pylori* IgG levels), and food-liking databases, incorporating Mendelian Randomization Phenome-Wide Association Study (MR-PheWAS) methodologies to identify phenotypes potentially influencing GC risk.

Further investigations explored the intricate relationships between blood markers, gut microbiota, and GC risk. This phase involved the analysis of various metabolites, immune cell populations, lipid profiles, inflammatory proteins, and microbiota through two-sample MR techniques.

The final phase of the study comprised transcriptomic and epigenetic analyses. TCGA and GTEx databases were employed for differential gene expression analysis, while the eQTLGen dataset was utilized for eQTL analysis through SMR. The integration of Differentially Expressed Genes (DEGs) and eQTLs facilitated the identification of potential protective and risk genes for GC.

Please refer to the Supplementary method and Supplementary Table for detailed study design and data resources.

## Results

### Unraveling the global epidemiology of GC

#### Global disparities in GC analysis of DALYs rates and trends

**Figure 2:**
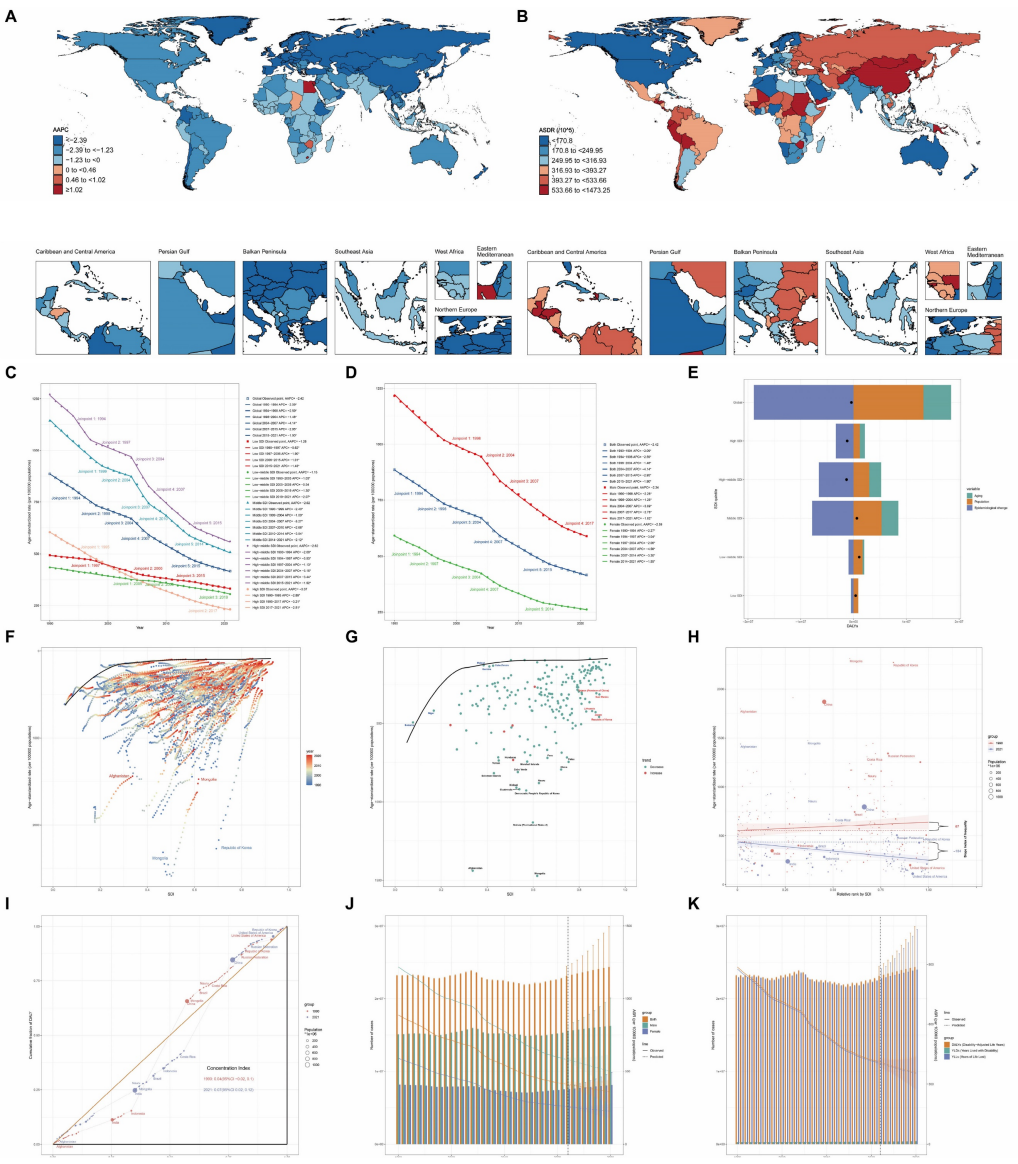
** Global Burden of Disease (GBD) Analysis Results for Gastric Cancer (GC).** World maps depict **(A)** the Average Annual Percent Change (AAPC) of age-standardized disability-adjusted life year rates (ASDR) from 1990 to 2021 and **(B)** ASDR in 2021. ASDR trends from 1990 to 2021, analyzed using Joinpoint regression model, are shown **(C)** stratified by Socio-demographic Index (SDI) regions and by **(D)** gender. **(E)** Decomposition analysis of variations in GC Disability-Adjusted Life Years (DALYs) between 1990 and 2021 accounts for changes in age structure, population size, and epidemiological factors. **(F, G)** Frontier analysis based on SDI and ASDR from 1990 to 2021 is presented. Health inequalities in 204 countries and regions are illustrated using **(H)** the Slope Index of Inequality (SII) for absolute inequalities and **(I)** the Concentration Index for relative inequalities. Predictions using the Bayesian Age-Period-Cohort (BAPC) model show **(J)** GC DALY numbers and Age-Standardized Rates (ASR) by gender from 2022 to 2030, and **(K)** GC DALY, Years Lived with Disability (YLD), and Years of Life Lost (YLL) numbers and ASRs from 2022 to 2030.

Figure 2A illustrates the global landscape of ASDR trends due to GC from 1990 to 2021. The majority of countries and regions worldwide exhibited varying degrees of decline in the average annual percentage change (AAPC) of ASDR. Notably, the Republic of Korea (-5.06% [95% uncertainty interval (UI), -5.32 to -4.79]), Maldives (-4.50% [95% UI, -4.76 to -4.23]), and Singapore (-4.36% [95% UI, -5.14 to -3.57]) demonstrated the most substantial reductions, ranking as the top three countries with the largest decreases in AAPC. In contrast, only five nations—Egypt (1.27%), Lesotho (1.17%), Zimbabwe (0.72%), Chad (0.33%), and Honduras (0.19%)—exhibited an upward trend in AAPC during the same period (Supplementary Table S1). Figure 2B depicts the ASDR rankings for 2021, revealing substantial global disparities. The countries with the highest ASDR (per 100,000 population) were Mongolia (1473.25), Afghanistan (1438.82), and Bolivia (1131.23). Conversely, Kuwait (86.83), Morocco (95.18), and Nigeria (96.00) reported the lowest ASDR values (Supplementary Table S2).

#### Comparative analysis of global GC burden trends by gender and SDI

Joinpoint regression analysis unveiled significant changes in the ASDR of GC over time. SDI stratification revealed evolving patterns of GC burden. In 1990, the High-middle SDI region reported the highest ASDR (1271.16), while the Low-middle SDI region had the lowest (434.36). By 2021, the High-middle SDI region (559.68) maintained the highest ASDR, whereas the High SDI region (231.61) reported the lowest. From 1990 to 2021, ASDR reductions were more substantial in the High SDI, High-middle SDI, and Middle SDI regions, with decreases of -3.07%, -2.62%, and -2.62%, respectively. In contrast, the Low-middle SDI and Low SDI regions experienced less pronounced declines of -1.15% and -1.26%, respectively (Figure 2C).

Figure 2D illustrates the ASDR per 100,000 population from 1990 to 2021, with notable joinpoints identified for males, females, and the overall population. The overall population ASDR exhibited a continuous decline with significant joinpoints in 1998, 2004, 2007, and 2015. The annual percentage change (APC) varied across time periods: -2.09% before 1994, -2.59% between 1994-1998, -1.48% between 1998-2004, -4.14% from 2004-2007, -2.95% from 2007-2015, and -1.90% from 2015-2021. The AAPC over the entire period was -2.42%. Gender-specific analysis revealed an AAPC of -2.34% for males and -2.59% for females over the study period. The ASDR for males (1216.22 in 1990; 588.45 in 2021) consistently exceeded that of females (591.03 in 1990; 261.96 in 2021).

#### Drivers of GC epidemiology: population growth, aging, and epidemiologic changes

A decomposition analysis of raw DALYs by population, age structure, and epidemiologic changes was conducted to elucidate the forces shaping GC epidemiology over the past three decades (Figure 2E and Supplementary Table S3). Globally, a net decline in GC DALYs was observed (represented by the black dot in Figure 2E, indicating the aggregate change contributed by all three components). From 1990 to 2021, while aging and population growth contributed to increases in DALYs by 1287.46% and 3251.44% respectively, substantial epidemiological changes in GC (contributing to a 4638.90% decrease) counterbalanced these increases, resulting in an overall reduction in GC-attributable DALYs. Regional analysis revealed divergent patterns. In High and High-middle SDI regions, raw DALYs decreased, predominantly due to epidemiological changes, with reductions of 279.95% and 495.71% respectively. Conversely, Middle, Low-middle, and Low SDI regions experienced increases in raw DALYs, primarily driven by population growth, with contributions of 832.4%, 158.89%, and 224.91% respectively.

#### Disparities in GC management efficiency

To quantify potentially achievable improvements in GC ASDR relative to a country's development status, a frontier analysis based on ASDR and SDI was constructed using data from 1990 to 2021 (Figure 2F). The frontier line delineates countries and territories with the lowest ASDR (optimal performers) given their SDI. The distance from this frontier, termed the effective difference, represents the gap between a country's observed and potentially achievable ASDR—a disparity that could potentially be mitigated based on the country or territory's sociodemographic resources.

Mongolia and the Republic of Korea are highlighted as exemplars of divergent trajectories. In 1990, these nations had the highest ASDRs globally, with values of 2315.52 and 2272.91 respectively. By 2021, the Republic of Korea had markedly reduced its ASDR to 457.74. However, Mongolia maintained its position as the country with the highest GC ASDR globally, with a value of 1473.25 in 2021, followed closely by Afghanistan at 1438.82 (Supplementary Table S4).

The effective difference from the frontier for each country and territory was calculated using 2021 ASDR and SDI data (Figure 2G and Supplementary Table S5). Among lower SDI countries (SDI < 0.5), Somalia, Niger, Malawi, Gambia, and Côte d'Ivoire demonstrated the smallest effective differences in GC management, ranging from 0 to 18.74. In contrast, higher SDI countries (SDI > 0.85), including the Republic of Korea, Japan, Lithuania, San Marino, and Taiwan (Province of China), exhibited significantly larger effective differences, ranging from 217.93 to 369.00.

A detailed assessment, irrespective of SDI, identified Mongolia, Afghanistan, Bolivia, the Democratic People's Republic of Korea, Guatemala, and several other nations (denoted in black in Figure 2G) among the top fifteen countries with the largest discrepancies between expected and actual performance in GC management. These countries exhibited effective differences ranging from 624.44 to 1376.44, underscoring substantial opportunities for improvement in GC outcomes relative to their sociodemographic resources.

#### Absolute inequality and relative inequality in GC burden

Figures 2H and 2I illustrate the top countries and regions with the highest ASDR in five SDI categories for 1990 and 2021, alongside the five most populous nations and regions.

The slope index of inequality, depicted in Figure 2H, quantifies the absolute inequality in GC burden across countries. In 1990, the index value of 87 indicated substantial absolute inequality, with higher ASDR observed in countries with higher SDI ranks. By 2021, the index shifted to -184, signifying a reversal in this trend, with higher GC ASDR now more prevalent in countries with lower SDI ranks.

Figure 2I presents the concentration index analysis, elucidating the relative inequality in GC burden among countries. The concentration index in 1990 was 0.04 (95% confidence interval [CI]: -0.02, 0.1), suggesting minimal inequality in GC burden distribution across countries with varying SDI. By 2021, the index increased to 0.07 (95% CI: 0.02, 0.12), indicating a slight increase in relative inequality. However, the difference in p-value (0.71) did not reach statistical significance, implying no substantial change in relative inequality between 1990 and 2021.

#### Temporal trends in DALYs and ASDR: 1990-2030

This study presents trends in DALYs, years lived with disability (YLDs), years of life lost (YLLs), and gender-specific data for GC globally from 1990 to 2030, encompassing historical data (1990-2021) and projections (2022-2030).

As illustrated in Figure 2J and Figure 2K, the total DALYs attributed to GC fluctuated between 1990 and 2021, peaking around 2004 (23.8 million DALYs). A general decline ensued from 2005, reaching approximately 22.9 million DALYs in 2014. However, projections indicate a concerning upward trend from 2015, with estimates reaching 24.3 million DALYs by 2030. YLDs, representing non-fatal disease burden, demonstrate a steady increase throughout the study period. YLLs, indicative of premature mortality, exhibit wave-like fluctuations from 1990 to 2030, similar to the overall DALYs trend.

Gender-specific DALYs trends mirror the overall population pattern.

The global ASDR for GC shows a consistent decline over the 41-year period from 1990 to 2030 (from 886.24 to 358.42 per 100,000 population). The ASR for YLDs remains relatively stable with a slight decrease (from 9.72 to 5.35). The marked decline in ASR for YLLs emerges as the primary driver of the overall ASDR reduction (from 876.50 to 353.17).

Throughout the entire period (1990-2030), males exhibit substantially higher ASDR for GC compared to females. In 1990, the male ASDR was 1216.21 per 100,000 population, while the female ASDR was 591.01, indicating more than twice the burden in males. This disparity is projected to persist, with male ASDR estimated at 500.50 in 2030, compared to 226.69 for females. Despite the difference in magnitude, both genders demonstrate similar downward trends in ASDR for GC.

### Potential protective and risk factors for GC

#### Clinical characteristics potentially associated with GC

Table 1 presents a complete analysis of PSM results, comparing various clinical variables between control and case groups before and after matching. A 1:10 PSM was performed between the disease group and the control group to balance age and gender distributions. The standardized mean difference (Std. Mean Diff.) served as the primary indicator of PSM effectiveness.

**Figure 3:**
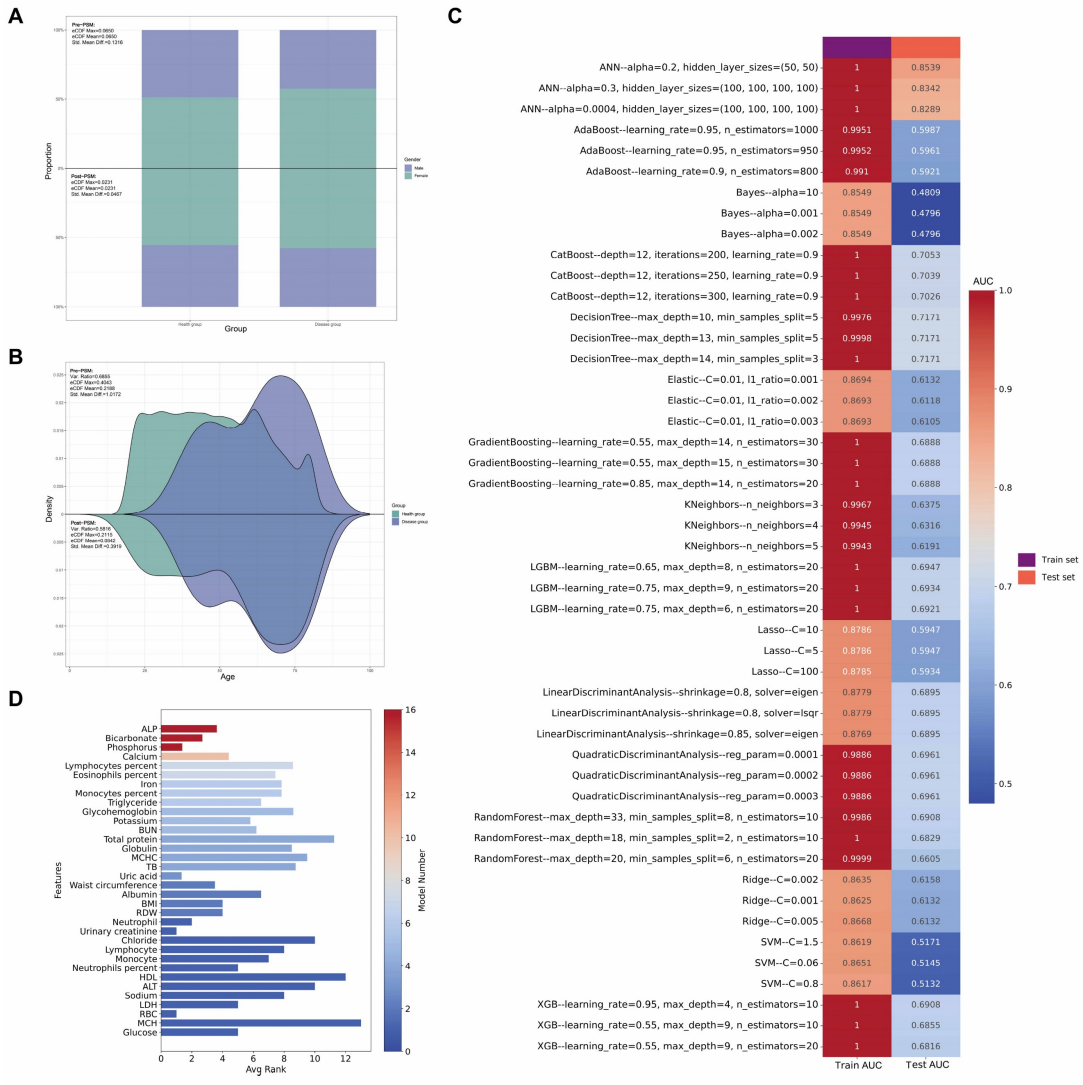
** Multi-panel visualization of propensity score matching outcomes and machine learning model performance. (A)** Comparative analysis of gender distribution pre- and post-propensity score matching (PSM), with accompanying statistical metrics demonstrating matching efficacy. **(B)** Density estimation plots depicting the age distribution before and after PSM implementation, with corresponding balance diagnostics. **(C)** Heatmap showcasing the top 3 optimal parameters for 16 machine learning models, selected based on Area Under the Curve (AUC) values in the test set. **(D)** Bar chart ranking variables by their frequency of occurrence and importance across all 16 machine learning models.

For sex, the Std. Mean Diff. decreased from 0.1316 to 0.0467 post-matching (Figure 3A). Similarly, for age, the Std. Mean Diff. decreased from 1.0172 to 0.3919 post-matching (Figure 3B). These results demonstrate the effectiveness of PSM in reducing age and gender bias, facilitating more accurate comparisons of other variables. Prior to matching, significant differences (p<0.05) were observed between cases and controls in age, urinary albumin, alanine aminotransferase (ALT), alkaline phosphatase (ALP), iron, osmolality, weight, waist circumference, eosinophils percentage, red blood cell count (RBC), hemoglobin, hematocrit, mean corpuscular hemoglobin (MCH), mean corpuscular hemoglobin concentration (MCHC), red cell distribution width (RDW), and glycohemoglobin. Post-matching analysis of 26 cases and 260 controls revealed a reduction in differences for all previously significant variables. However, several variables maintained statistically significant differences: urinary albumin (p = 0.015), calcium (p = 0.027), iron (p = 0.012), phosphorus (p = 0.013), chloride (p = 0.006), waist circumference (p = 0.023), hemoglobin (p = 0.015), hematocrit (p = 0.022), MCHC (p = 0.049), RDW (p = 0.005), and platelet count (p = 0.045).

Logistic regression analysis of the propensity score-matched data revealed several clinical characteristics potentially associated with GC risk (Supplementary Table S6). Higher levels of iron, weight, waist circumference, hemoglobin, hematocrit, MCH, and MCHC were associated with a reduced risk of GC (all p<0.05). Conversely, increased levels of phosphorus, chloride, eosinophils percentage, RDW, and platelet count were associated with an increased risk (all p<0.05). Subsequent multivariable logistic regression analysis, which included variables with p<0.05 from the univariable analysis, identified two factors that remained significantly associated with GC risk. Eosinophils percentage (odds ratio [OR] range: 1.26, 95% CI: 1.03-1.54, p = 0.022) and RDW (OR: 1.71, 95% CI: 1.11-2.64, p = 0.015) demonstrated independent associations with increased GC risk.

In addition to using logistic regression, we employed an extensive array of machine learning techniques to elucidate the association between clinical characteristics and GC. Our methodology encompassed 16 distinct models. This multifaceted approach was designed to optimize the identification of clinical parameters associated with GC, as quantified by the Area Under the Curve (AUC) metric. Figure 3C illustrates the discriminative capacity of each model. Notably, 14 of the 16 models demonstrated robust performance, with AUC values ranging from 0.59 to 0.85. The Naive Bayes and Support Vector Machine (SVM) models, however, exhibited comparatively inferior discriminative abilities.

To ascertain the relative importance of clinical parameters, we utilized Shapley Additive exPlanations (SHAP) values, evaluating each characteristic across all 16 models with their optimized parameters in the test set. Figure 3D presents a bar chart of these findings, with the color gradient from red to blue indicating the frequency of inclusion for each clinical characteristic across the models. The x-axis represents the mean importance rank of each characteristic, with lower values denoting higher importance. Our analysis revealed that ALP, bicarbonate, and phosphorus emerged as the most significant parameters, while RBC, MCH, and glucose were identified as the least influential.

**Figure 4:**
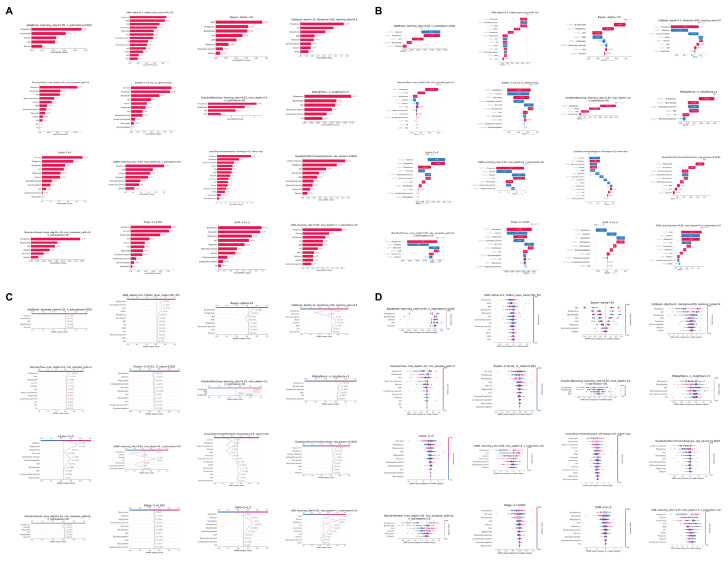
** Comprehensive model interpretability and feature contribution analysis. (A)** Bar charts detailing variables included in each model, their absolute SHAP (SHapley Additive exPlanations) values, and variable importance rankings. **(B)** Waterfall plots and **(C)** decision plots based on the test set, providing detailed insights into how individual variables influence the model's decision-making process in classifying samples as either GC or control. **(D)** Beeswarm plots visualizing the relationship between various clinical characteristics and GC risk in the test set.

Figure 4A provides a detailed breakdown of the absolute SHAP scores for each clinical characteristic across all 16 optimized machine learning models in the test set, with variables ranked in descending order based on their absolute SHAP scores. To elucidate the predictive mechanisms of each model, we employed waterfall plots (Figure 4B) to visualize the prediction process for a GC sample from the test set. These plots delineate the contributions of individual characteristics to the model's predicted value, f(x). The prediction is computed by aggregating the SHAP values of each characteristic, starting from a base value. The z-score normalized values of each variable's actual value in the sample are displayed to the left of each characteristic. A positive f(x) value indicates a prediction favoring GC, while a negative value suggests a prediction leaning towards the healthy population. To further quantify the risk prediction for GC samples in the test set, we constructed a decision plot (Figure 4C). This plot employs a methodology similar to the waterfall plots but incorporates a color-coded line at the apex, ranging from 0 (blue) to 1 (red), representing the predicted probability of GC classification. A value exceeding 0.5 indicates a model prediction of greater than 50% likelihood of GC. Our analysis revealed that 14 of the 16 machine learning models correctly classified the sample as GC with a probability exceeding 0.5, while the Linear Discriminant Analysis (LDA) and SVM models incorrectly categorized the GC sample as belonging to the healthy cohort.

In the end, the beeswarm plot (Figure 4D) offers a sophisticated visualization of the impact of individual clinical characteristics on the model's predictive output within the test set. This nuanced representation facilitates the identification of potential associations between the clinical parameters and GC. The plot reveals that elevated levels of certain biochemical markers, including ALP, phosphorus, calcium, eosinophil percentage, triglycerides, and potassium, may be correlated with an increased likelihood of GC. Conversely, diminished levels of bicarbonate, iron, monocyte percentage, and MCHC appear to be associated with a higher probability of GC.

#### Disease, food-liking phenotypes, gut microbiota composition and various blood-based biomarkers potentially associated with GC risk

We conducted two-sample MR analyses to elucidate potential risk factors and protective elements associated with GC. Our investigation encompassed a wide array of exposures, including disease phenotypes, food preferences, gut microbiota composition, and various blood-based biomarkers, utilizing GC genome-wide association study (GWAS) data as the outcome. All the results in this study do not exist pleiotropy and heterogeneity.

**Figure 5:**
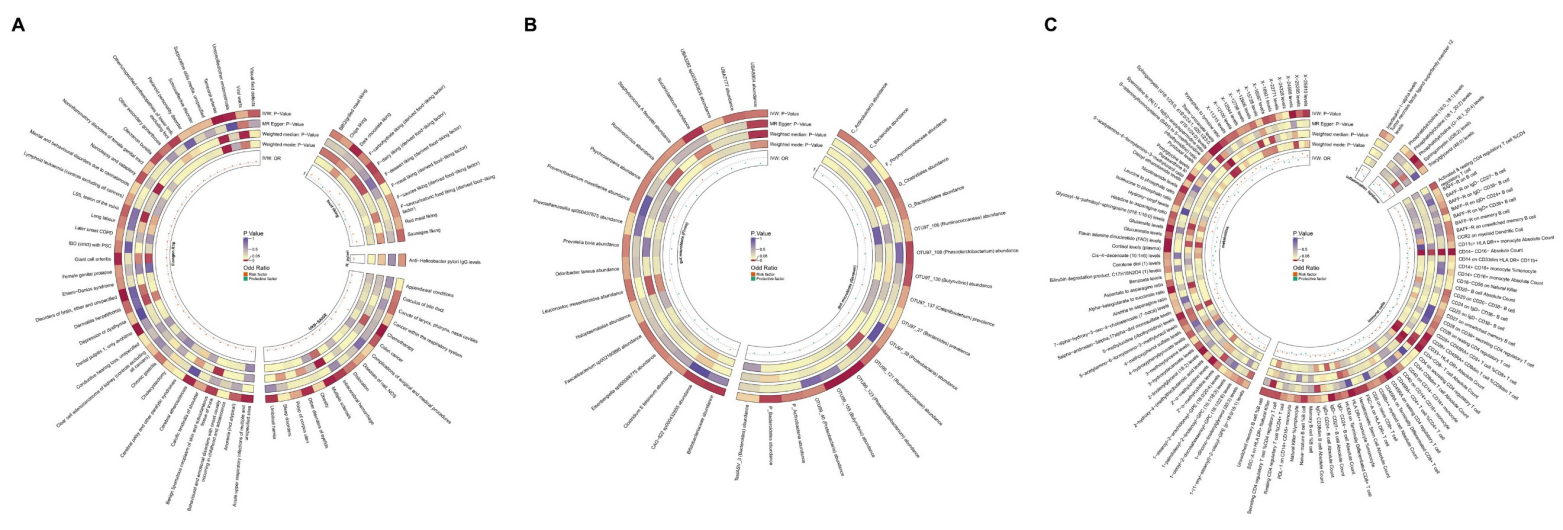
** Integrative circular visualization of multidimensional risk factors and molecular signatures associated with GC. (A)** Circular heatmap illustrating disease phenotypes and dietary preferences influencing GC risk. **(B)** Circular heatmap depicting microbial factors affecting GC risk. **(C)** Circular heatmap showing the impact of metabolites, immune cell populations, lipid species, and inflammatory proteins on GC risk.

Leveraging data from the FinnGen R10 GWAS (2,408 phenotypes), UK Biobank-SAIGE GWAS (783 phenotypes), *Helicobacter pylori* infection, and food liking (139 specific foods) databases, we identified some disease and behavioral phenotypes potentially linked to increased GC risk (OR: 1.03-2.80) (Figure 5A, Supplementary Table S7). Notable among these were anti-*Helicobacter pylori* IgG levels, chronic gastritis, obesity, depression or dysthymia, and biliary calculi, which have been previously implicated in GC pathogenesis. Our analysis of food preferences revealed 11 phenotypes significantly associated with GC risk. Dairy product preference exhibited the strongest positive correlation (OR: 2.42, p = 0.009), followed by preferences for sausages (OR: 1.76, p = 0.017), chips (OR: 1.60, p = 0.030), barbecued/grilled meat (OR: 1.51, p = 0.022), and red meat (OR: 1.39, p = 0.034). Carbohydrate (OR: 1.32, p = 0.024), dessert (OR: 1.19, p = 0.013), savory/caloric food (OR: 1.18, p = 0.027), and general meat (OR: 1.16, p = 0.049) preferences also showed positive associations. Conversely, preferences for dark chocolate (OR: 0.85, p = 0.004) and sauces (OR: 0.82, p = 0.028) were associated with reduced GC risk.

Our analysis of gut microbiota revealed distinct associations with GC risk across German and Finnish cohorts. In the German cohort, such as *Phascolarctobacterium* (OTU99_123) and *Butyrivibrio* (OTU99_155) demonstrated strong protective associations (OR: 0.89, p = 0.002; OR: 0.94, p = 0.002, respectively), while *Ruminococcaceae* (OTU99_121) showed the strongest risk association (OR: 1.11, p = 0.018). The Finnish cohort analysis identified UBA8904 as having the most significant protective association (OR: 0.51, p = 0.021), whereas *Faecalibacterium* sp002160895 exhibited the strongest risk association (OR: 1.39, p = 0.020) (Figure 5B, Supplementary Table S8).

Among plasma metabolites and metabolite ratios, 56 markers showed significant associations with GC risk (Figure 5C, Supplementary Table S9). Cortisol (OR: 0.85, p = 0.001), glutamate (OR: 0.85, p = 0.002), and flavin adenine dinucleotide (OR: 0.89, p < 0.001) levels demonstrated strong protective effects. In contrast, benzoate (OR: 1.11, p = 0.044), nicotinamide (OR: 1.15, p = 0.006), and the leucine to phosphate ratio (OR: 1.13, p = 0.021) were associated with increased GC risk.

The analysis of immune cell markers revealed 51 significant associations with GC risk (Figure 5C, Supplementary Table S9). Natural Killer %lymphocyte exhibited a protective effect (OR: 0.97, p = 0.022). Conversely, CD4-CD8- T cell absolute count (OR: 1.12, p = 0.001), CD45RA- CD4+ T cell %CD4+ T cell (OR: 1.07, p = 0.010), and hematopoietic stem cell absolute count (OR: 1.04, p = 0.008) were positively associated with GC risk.

In the plasma lipidome analysis, five significant associations were identified (Figure 5C, Supplementary Table S9). Phosphatidylcholine (16:0_18:1) (OR: 0.91, p = 0.044), phosphatidylcholine (O-16:1_20:4) (OR: 0.93, p = 0.001), and sphingomyelin (d38:2) (OR: 0.93, p = 0.010) levels demonstrated protective effects against GC. Conversely, phosphatidylcholine (18:1_20:2) (OR: 1.05, p = 0.033) and triacylglycerol (48:0) (OR: 1.07, p = 0.037) levels showed positive associations with GC risk.

The analysis of inflammatory markers revealed two significant associations with GC risk (Figure 5C, Supplementary Table S9). Interleukin-1-alpha levels were associated with increased risk (OR: 1.09, p = 0.027), while tumor necrosis factor ligand superfamily member 12 levels exhibited a protective effect (OR: 0.92, p = 0.038).

#### Genes potentially associated with GC risk

In our study, we conducted a systematic investigation into the genetic underpinnings of GC risk, employing a multi-tiered approach to categorize candidate genes into three evidence levels, with level 1 representing the strongest evidence.

**Figure 6:**
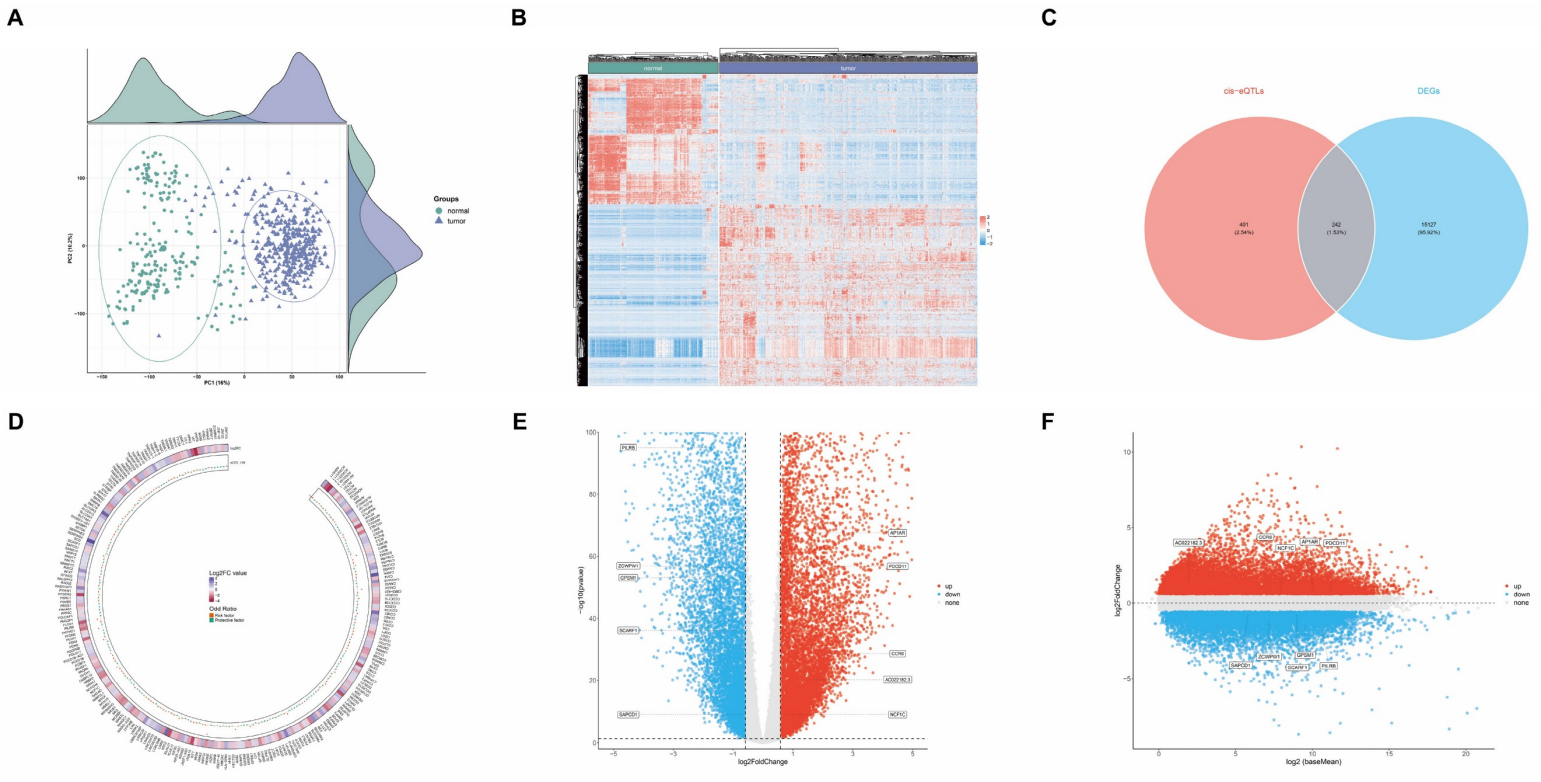
** Comprehensive multi-platform genomic analysis integrating transcriptomic profiles and genetic architecture of GC. (A)** Principal Component Analysis (PCA) plot showing the distribution of samples from The Cancer Genome Atlas (TCGA) and Genotype-Tissue Expression (GTEx) databases. **(B)** Heatmap depicting gene expression patterns in GC versus healthy tissues. **(C)** Venn diagram illustrating the intersection of Differentially Expressed Genes (DEGs) from bioinformatics analysis with expression Quantitative Trait Loci (eQTL) results from Summary-data-based Mendelian Randomization (SMR) analysis. **(D)** Circular heatmap providing insights into the relationships among 242 gene fold change, gene expression, and GC risk. **(E)** Volcano plot demonstrating the relationship between colocalized gene fold change and p-value. **(F)** MA plot delineating the relationship between colocalized gene mean expression levels and log-fold changes.

We initiated our analysis by examining differential gene expression in GC using datasets from TCGA and GTEx databases. Our cohort comprised 621 samples, including 413 GC tissue samples and 208 healthy tissue samples. The principal component analysis (PCA) plot in Figure 6A illustrates the distribution of these samples, while Figure 6B presents a heatmap depicting their gene expression patterns. Employing a threshold of 1.5-fold change in gene expression, we identified 15,369 genes exhibiting significant differential expression between GC and healthy tissues (p<0.05), categorized as evidence level 3 (Supplementary Table S10).

To further elucidate genetic associations with GC risk, we conducted SMR analyses. Using cis-expression quantitative trait loci (cis-eQTL) GWAS data from the eQTLGen database as exposure and GC GWAS as outcome, we identified 643 genes associated with GC risk (Supplementary Table S11). The intersection of these two datasets yielded 242 genes potentially linked to GC risk (Figure 6C and Supplementary Table S12), classified as evidence level 2. The circular heatmap providing insights into the relationships among 242 gene fold change, gene expression, and GC risk (Figure 6D).

To further validate the reliability of these 242 genes associated with GC risk, we employed colocalization analysis. This approach aims to determine whether a common causal variant is responsible for both gene expression and GC risk within a specific genomic region. We utilized a Bayesian statistical framework to calculate posterior probabilities for various hypotheses regarding the relationship between gene expression and disease risk. Our analysis focused on the posterior probability of hypothesis 4 (PP.H4), which represents the likelihood that both gene expression and GC risk share a common causal variant. We established a threshold of PP.H4 > 0.5 to indicate significant colocalization, suggesting a greater than 50% probability that the same causal variant influences both gene expression and GC risk in the region of interest.

Our colocalization analysis identified 10 genes with significant evidence of shared causal variants between eQTLs and GC risk. These genes, namely *AC022182.3*, *AP1AR*, *CCR6*, *GPSM1*, *NCF1C*, *PDCD11*, *PILRB*, *SAPCD1*, *SCARF1*, and *ZCWPW1*, were classified as evidence level 1, representing the strongest level of evidence in our study (Supplementary Table S13). The volcano plot (Figure 6E) depicts the relationship between these 10 genes fold change and p-value, while the MA plot (Figure 6F) illustrates the relationship between the average expression of these genes in the samples and their fold change.

**Figure 7:**
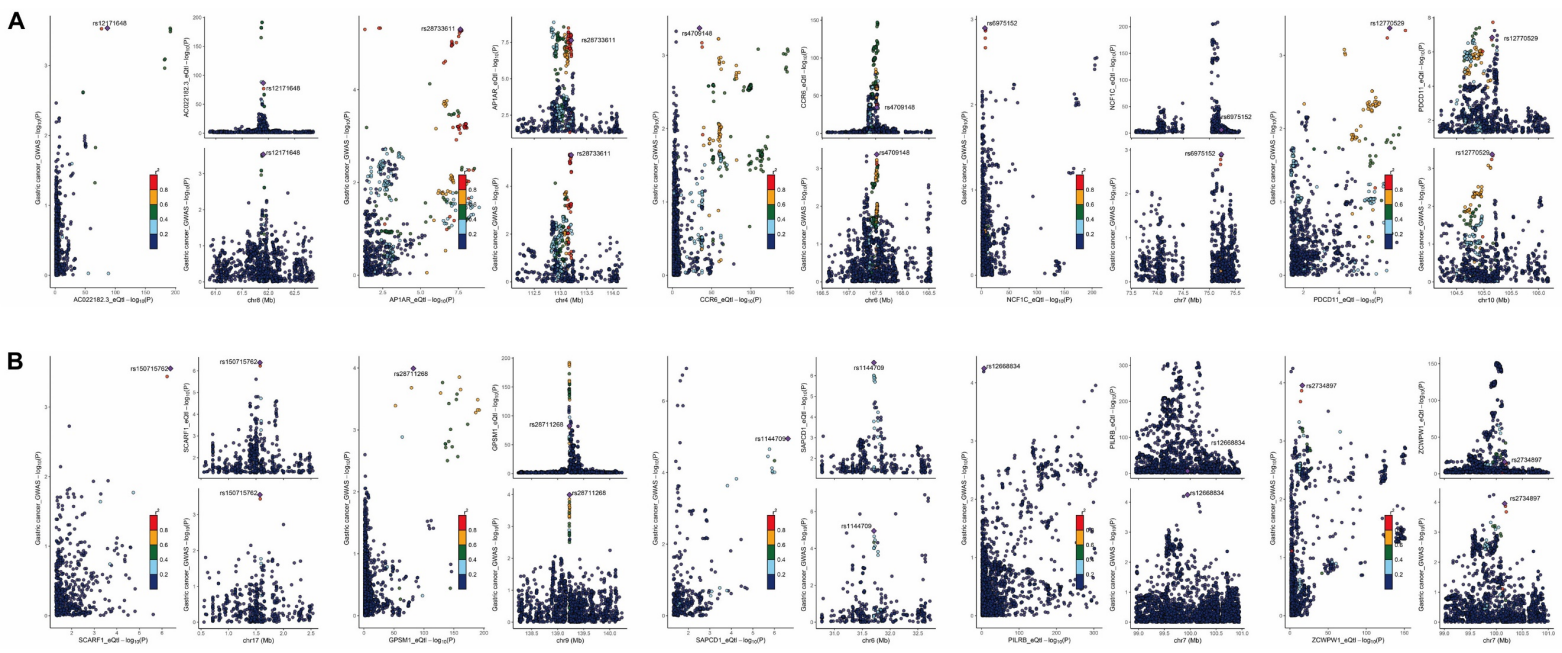
** Colocalization analysis revealing tissue-specific gene regulation patterns in GC. (A)** Colocalization plots depicting genes upregulated in GC tissue. **(B)** Colocalization plots illustrating genes downregulated in GC tissue.

The colocalization plot depicting genes upregulated (Figure 7A) and downregulated (Figure 7B) in GC tissue, with the lead single nucleotide polymorphism (SNP) labeled and other SNPs color-coded according to their linkage disequilibrium (LD) with the lead SNP.

## Discussion

This study provides an in-depth analysis of the epidemiological trends of GC from 1990 to 2030, elucidating associated risk factors and genetic markers. Our findings reveal substantial global disparities in GC burden, with a notable decline in ASDR observed in most countries. Conversely, a subset of nations experienced increases in ASDR. Furthermore, projections indicate a concerning upward trajectory in total DALYs from 2015 onwards, with estimates reaching 24.3 million DALYs by 2030, underscoring persistent challenges in GC management. The study identifies a constellation of factors potentially influencing GC risk, including clinical characteristics, disease phenotypes, dietary preferences, microbiota composition, metabolite profiles, immune cell populations, lipid profiles, inflammatory proteins, and genetic markers. The insights derived from this extensive analysis have the potential to inform public health strategies, guide clinical practice in risk assessment and early detection, and direct future research endeavors in GC prevention and treatment. As we confront the ongoing challenge of mitigating the global burden of GC, this multifaceted approach offers a novel paradigm for understanding and addressing complex diseases in the era of precision medicine and big data analytics.

Our analysis of global GC burden trends reveals a general decline in ASDR from 1990 to 2021, with significant reductions observed in countries such as the Republic of Korea, Maldives, and Singapore. This decline can be attributed to advancements in medical technology, enhanced healthcare infrastructure, and efficacious public health interventions targeting GC risk factors. However, countries including Egypt, Lesotho, and Zimbabwe exhibited increasing ASDR, suggesting persistent disparities in healthcare access, socioeconomic development, and cancer control strategies 6, 23-27. Our examination of GBD data unveils a complex pattern of GC epidemiology from 1990 to 2030. While the ASDR for GC demonstrates a consistent global decline, projections indicate a concerning upward trend in DALYs from 2015 onwards, reaching an estimated 24.3 million DALYs by 2030. This paradoxical trend underscores the evolving nature of GC burden, likely influenced by population growth and aging, despite improvements in prevention and treatment modalities.

The substantial reduction in ASDR, primarily driven by decreases in YLLs, suggests improvements in early detection and treatment efficacy. However, the persistent gender disparity, with males exhibiting consistently higher ASDR, highlights the need for targeted interventions and research into sex-specific risk factors and biological mechanisms 28, 29. The shift in the slope index of inequality from 87 in 1990 to -184 in 2021 indicates a reversal in the association between SDI and GC burden, with lower SDI countries now bearing a disproportionate burden. This trend aligns with previous studies highlighting the growing cancer burden in low- and middle-income countries 30, 31. The frontier analysis provides valuable insights into the potential for improvement in GC management across different SDI levels. The significant gaps between observed and potentially achievable DALYs in countries such as Mongolia and Afghanistan highlight opportunities for targeted interventions and resource allocation.

Our propensity score-matched analysis of NHANES data unveils several clinical characteristics associated with GC risk. Notably, the identification of eosinophil percentage and RDW as independent risk factors in multivariable analysis is particularly intriguing. Elevated eosinophil counts have previously been linked to gastric inflammation and precancerous lesions, while increased RDW has been associated with poorer survival in various cancers, potentially reflecting underlying inflammation or nutritional deficiencies 32-38. The consistent identification of ALP, bicarbonate, and phosphorus levels associated with GC by machine learning models contributes to the growing body of evidence linking metabolic alterations to cancer development. Elevated ALP and phosphorus levels correlating with increased GC risk may indicate underlying liver dysfunction or bone metastases, while lower bicarbonate levels might suggest metabolic acidosis, a condition associated with cancer progression 39-44. These findings underscore the intricate interplay between systemic metabolic perturbations and GC development, suggesting potential avenues for risk assessment and early detection. However, the observational nature of these associations necessitates further research to establish causality and elucidate underlying mechanisms.

Our MR analyses provide evidence for causal relationships between various disease phenotypes, dietary preferences, and GC risk. The identification of *Helicobacter pylori* infection, chronic gastritis, obesity, and depression as potential risk factors aligns with previous studies 45-49. The findings on dietary preferences offer valuable insights for public health interventions. The positive associations between GC risk and preferences for dairy, processed meats (sausages), and high-calorie foods (chips, BBQ/grilled meat) corroborate existing evidence on the role of diet in GC etiology 50. These results strengthen the case for dietary modifications as a preventive strategy against GC. Conversely, the protective association of dark chocolate preference with GC risk is intriguing and may be related to its high polyphenol content, known for anti-inflammatory and antioxidant properties 51-54. This finding, if confirmed in further studies, could inform dietary recommendations for GC prevention.

Our MR analyses of gut microbiota and blood-based biomarkers unveil an intricate network of potential risk and protective factors for GC. The identification of specific bacterial taxa associated with GC risk, notably the protective effect of *Phascolarctobacterium* and the risk association of *Ruminococcaceae* species, contributes to the expanding body of evidence implicating the gut microbiome in GC pathogenesis 55-59. These findings suggest promising avenues for microbiome-based risk stratification and therapeutic interventions. The associations between plasma metabolites and GC risk provide insights into the metabolic alterations that may precede or accompany GC development. Our study corroborates previous research by demonstrating that elevated nicotinamide levels correlate with increased GC risk 60. Immune cell analyses, particularly the protective association of Natural Killer cell percentage and the risk association of specific T cell subsets, underscore the critical role of immune surveillance in GC development. These results complement existing knowledge on immune dysfunction in cancer and suggest potential immunological biomarkers for GC risk assessment.

Our most robust finding is the identification of 10 genes (*AC022182.3*, *AP1AR*, *CCR6*, *GPSM1*, *NCF1C*, *PDCD11*, *PILRB*, *SAPCD1*, *SCARF1*, and *ZCWPW1*) showing significant evidence of shared causal variants between eQTLs and GC risk. This colocalization suggests a potential causal relationship between the expression of these genes and GC risk. Among these colocalized genes, we identified both potential risk-increasing (e.g., *AC022182.3*, *NCF1C*, *SAPCD1*) and protective (e.g., *AP1AR*, *CCR6*, *SCARF1*) genes, underscoring the complex nature of genetic contributions to GC risk.

Our findings both corroborate and extend previous research on GC genetics. For instance, we identified *CCR6* as significantly upregulated in GC tissues and associated with a protective effect against GC risk. This aligns with previous research showing *CCR6*'s role in immune cell recruitment and its potential involvement in anti-tumor responses 61. However, our finding of a protective effect contrasts with some studies suggesting *CCR6* promotes tumor progression, highlighting the complex and context-dependent role of this gene in cancer 62, 63. Similarly, we found *SCARF1* to be downregulated in GC tissues and strongly associated with a protective effect against GC risk. This is consistent with its known function as a multifaceted scavenger receptor that plays a critical role in both innate and adaptive immunity. It facilitates the uptake and processing of antigens, activates inflammatory pathways, and is essential for the clearance of apoptotic cells, thereby maintaining immune homeostasis 64, 65.

While our study presents an exhaustive analysis of GC epidemiology and risk factors, several limitations warrant consideration. Firstly, the GBD projections, although based on sophisticated modeling techniques, are inherently subject to uncertainties, particularly in rapidly evolving environments or in the face of unforeseen global events. Future research should focus on continuously updating these projections with the most recent data and refining modeling methodologies to enhance predictive accuracy. Secondly, despite utilizing the nationally representative NHANES database to minimize selection bias, the relatively small number of GC cases (n=26) represents a notable limitation that may affect the generalizability of our findings. Although we implemented multiple methodological approaches to address this limitation - including PSM to reduce potential confounding, ADASYN sampling for training set balancing, and validation through 16 different machine learning models - the fundamental constraint of sample size necessitates cautious interpretation of our results. This limitation is inherent to the use of public databases where GC cases may be underrepresented. Moreover, the cross-sectional nature of NHANES data precludes the establishment of causal relationships between the identified factors and GC. To enhance the robustness of our findings, future research should focus on prospective cohort studies specifically designed to evaluate the clinical indicators identified in our analysis. Thirdly, while our MR analyses provide compelling evidence for causal relationships, it is crucial to acknowledge that these analyses rely on several assumptions that may not always hold true in complex biological systems. Prospective cohort studies are necessary to validate the predictive value of the identified clinical, microbial, and molecular biomarkers for GC risk assessment. Fourthly, our gene expression and genetic analyses, although comprehensive, are based on bulk tissue data, which may obscure important cell-type-specific effects. Lastly, another important limitation stems from the utilization of multiple databases representing different populations (U.S., Finnish, British, and German cohorts). While these databases provide robust, well-validated data, the heterogeneity of study populations may influence the generalizability of our findings across different ethnic and geographical groups. Future multi-ethnic, multi-center prospective studies are needed to validate these findings across diverse populations and to better understand potential population-specific variations in the identified associations.

## Conclusion

This study provides a multifaceted view of GC epidemiology and risk factors, integrating global trends with novel insights into clinical, microbial, blood-based, genetic risk factors. Our findings highlight the complex and evolving nature of GC burden, the identification of novel risk factors offers potential avenues for improved risk stratification and targeted interventions. As we move forward, a concerted effort involving epidemiologists, clinicians, molecular biologists, and public health professionals will be crucial to translate these findings into effective strategies for GC prevention, early detection, and treatment. By addressing the multifaceted nature of GC risk, we can work towards reducing the global burden of this devastating disease.

## Supplementary Material

Supplementary methods and figure.

Supplementary tables.

## Figures and Tables

**Table 1:** Comparison of clinical characteristics between case and control groups before and after PSM

	Pre-PSM			Post-PSM		
Variable	Controls, N = 31,868^1^	Cases, N = 26^1^	p-value^2^	Controls, N = 260^1^	Cases, N = 26^1^	p-value^2^
Sex						
Female	16,313.00 (51.19%)	15.00 (57.69%)	0.507	144.00 (55.38%)	15.00 (57.69%)	0.821
Male	15,555.00 (48.81%)	11.00 (42.31%)		116.00 (44.62%)	11.00 (42.31%)	
Age	47.000 (33.000, 61.000)	65.000 (49.250, 76.000)	<0.001	62.500 (42.000, 72.000)	65.000 (49.250, 76.000)	0.172
Urinary albumin	8.000 (4.200, 16.600)	19.100 (9.425, 34.700)	<0.001	10.150 (4.600, 21.175)	19.100 (9.425, 34.700)	0.015
Urinary creatinine	113.000 (64.000, 171.000)	100.000 (76.250, 153.750)	0.812	93.000 (54.000, 169.500)	100.000 (76.250, 153.750)	0.538
Albumin	42.000 (40.000, 44.000)	41.500 (38.000, 44.000)	0.589	41.000 (39.000, 43.000)	41.500 (38.000, 44.000)	0.411
ALT	20.000 (15.000, 28.000)	16.000 (12.000, 20.750)	0.023	19.000 (14.000, 27.000)	16.000 (12.000, 20.750)	0.180
AST	22.000 (18.000, 27.000)	22.000 (18.500, 25.750)	0.985	20.000 (17.000, 26.000)	22.000 (18.500, 25.750)	0.486
ALP	68.000 (56.000, 83.000)	85.000 (62.250, 96.000)	0.012	73.000 (58.000, 90.250)	85.000 (62.250, 96.000)	0.149
BUN	4.640 (3.570, 5.710)	5.355 (3.660, 7.943)	0.124	5.360 (4.280, 6.430)	5.355 (3.660, 7.943)	0.852
Calcium	2.350 (2.275, 2.400)	2.363 (2.300, 2.400)	0.318	2.325 (2.250, 2.375)	2.363 (2.300, 2.400)	0.027
Cholesterol	4.913 (4.241, 5.637)	4.720 (4.183, 5.314)	0.329	4.810 (4.157, 5.637)	4.720 (4.183, 5.314)	0.564
Bicarbonate	25.000 (24.000, 27.000)	25.000 (23.000, 26.000)	0.546	25.000 (24.000, 27.000)	25.000 (23.000, 26.000)	0.227
Creatinine	74.260 (62.760, 88.400)	76.465 (66.743, 92.820)	0.536	75.140 (63.650, 90.390)	76.465 (66.743, 92.820)	0.779
GGT	20.000 (14.000, 31.000)	22.500 (12.250, 30.250)	0.714	22.000 (15.000, 31.000)	22.500 (12.250, 30.250)	0.463
Glucose	5.110 (4.720, 5.720)	5.410 (4.885, 6.095)	0.078	5.270 (4.830, 5.880)	5.410 (4.885, 6.095)	0.296
Iron	82.000 (61.000, 105.000)	69.000 (40.250, 95.750)	0.015	80.500 (63.750, 109.000)	69.000 (40.250, 95.750)	0.012
LDH	134.000 (117.000, 155.000)	134.000 (116.000, 174.250)	0.589	148.000 (132.000, 174.500)	134.000 (116.000, 174.250)	0.110
Phosphorus	1.195 (1.066, 1.292)	1.243 (1.098, 1.388)	0.095	1.162 (1.033, 1.259)	1.243 (1.098, 1.388)	0.013
TB	10.260 (6.840, 13.680)	9.405 (6.840, 11.970)	0.463	8.550 (5.130, 10.260)	9.405 (6.840, 11.970)	0.248
Total protein	72.000 (69.000, 75.000)	71.000 (69.000, 75.500)	0.924	71.000 (68.000, 74.000)	71.000 (69.000, 75.500)	0.817
Triglyceride	1.332 (0.903, 2.032)	1.434 (1.005, 2.340)	0.557	1.282 (0.960, 1.843)	1.434 (1.005, 2.340)	0.506
Uric acid	315.200 (261.700, 374.700)	282.550 (223.075, 345.000)	0.122	321.200 (261.700, 382.175)	282.550 (223.075, 345.000)	0.073
Sodium	140.000 (138.000, 141.000)	141.000 (138.000, 142.000)	0.179	140.000 (138.000, 141.000)	141.000 (138.000, 142.000)	0.463
Potassium	4.000 (3.800, 4.200)	4.100 (3.800, 4.300)	0.132	4.000 (3.800, 4.300)	4.100 (3.800, 4.300)	0.455
Chloride	103.000 (101.000, 105.000)	104.000 (101.250, 105.000)	0.491	101.000 (100.000, 103.000)	104.000 (101.250, 105.000)	0.006
Osmolality	279.000 (276.000, 282.000)	282.500 (279.250, 284.750)	0.004	280.000 (277.000, 284.000)	282.500 (279.250, 284.750)	0.169
Globulin	29.000 (27.000, 33.000)	30.000 (27.000, 32.750)	0.699	30.000 (27.000, 33.000)	30.000 (27.000, 32.750)	0.792
Weight	78.700 (66.700, 93.200)	68.750 (62.550, 80.975)	0.010	76.250 (66.600, 88.725)	68.750 (62.550, 80.975)	0.055
Standing height	166.800 (159.600, 174.400)	162.150 (159.450, 170.650)	0.108	164.600 (157.300, 171.625)	162.150 (159.450, 170.650)	0.695
BMI	28.120 (24.420, 32.700)	26.500 (24.055, 30.758)	0.098	27.900 (25.000, 31.825)	26.500 (24.055, 30.758)	0.141
Waist circumference	97.800 (87.500, 108.800)	93.600 (85.175, 100.775)	0.044	99.500 (89.300, 110.200)	93.600 (85.175, 100.775)	0.023
WBC	6.900 (5.700, 8.400)	6.900 (5.725, 8.975)	0.885	7.000 (5.600, 8.900)	6.900 (5.725, 8.975)	0.913
Lymphocytes percent	30.500 (25.200, 36.300)	30.400 (24.500, 37.425)	0.765	30.250 (24.950, 37.550)	30.400 (24.500, 37.425)	0.890
Monocytes percent	7.700 (6.400, 9.100)	7.500 (6.125, 8.575)	0.567	7.800 (6.800, 9.500)	7.500 (6.125, 8.575)	0.233
Neutrophils percent	58.000 (51.500, 64.000)	56.700 (49.650, 60.900)	0.397	58.150 (49.900, 63.700)	56.700 (49.650, 60.900)	0.589
Eosinophils percent	2.300 (1.500, 3.500)	2.750 (2.200, 3.900)	0.047	2.400 (1.500, 3.700)	2.750 (2.200, 3.900)	0.071
Basophils percent	0.700 (0.500, 0.900)	0.750 (0.500, 1.000)	0.480	0.700 (0.600, 0.900)	0.750 (0.500, 1.000)	0.909
Lymphocyte	2.100 (1.700, 2.600)	2.250 (1.600, 2.575)	0.779	2.200 (1.700, 2.600)	2.250 (1.600, 2.575)	0.917
Monocyte	0.500 (0.400, 0.700)	0.500 (0.400, 0.700)	0.762	0.600 (0.475, 0.700)	0.500 (0.400, 0.700)	0.320
Neutrophil	4.000 (3.100, 5.100)	3.950 (2.750, 5.275)	0.863	3.900 (3.000, 5.500)	3.950 (2.750, 5.275)	0.773
RBC	4.680 (4.350, 5.030)	4.520 (4.255, 4.683)	0.043	4.640 (4.350, 4.953)	4.520 (4.255, 4.683)	0.105
Hemoglobin	14.100 (13.100, 15.100)	13.500 (12.375, 14.050)	0.001	13.850 (13.000, 14.700)	13.500 (12.375, 14.050)	0.015
Hematocrit	41.700 (38.700, 44.600)	40.500 (37.925, 41.675)	0.008	41.350 (38.875, 43.700)	40.500 (37.925, 41.675)	0.022
MCV	89.400 (86.000, 92.400)	89.000 (80.400, 91.125)	0.113	89.500 (86.100, 92.500)	89.000 (80.400, 91.125)	0.105
MCH	30.400 (29.000, 31.600)	29.200 (25.950, 30.800)	0.015	30.100 (28.800, 31.200)	29.200 (25.950, 30.800)	0.065
MCHC	33.900 (33.300, 34.500)	32.900 (32.425, 33.975)	<0.001	33.600 (32.975, 34.100)	32.900 (32.425, 33.975)	0.049
RDW	13.100 (12.500, 13.800)	14.300 (13.425, 16.050)	<0.001	13.550 (13.000, 14.100)	14.300 (13.425, 16.050)	0.005
Platelet	242.000 (205.000, 286.000)	256.500 (213.250, 305.250)	0.207	235.000 (197.000, 270.000)	256.500 (213.250, 305.250)	0.045
MPV	8.100 (7.500, 8.700)	8.400 (8.000, 9.025)	0.069	8.200 (7.700, 8.800)	8.400 (8.000, 9.025)	0.231
Glycohemoglobin	5.500 (5.200, 5.900)	5.750 (5.425, 6.225)	0.016	5.700 (5.300, 6.100)	5.750 (5.425, 6.225)	0.353
HDL	1.320 (1.090, 1.600)	1.385 (1.095, 1.653)	0.486	1.320 (1.110, 1.660)	1.385 (1.095, 1.653)	0.760

^1^n (%); Median (IQR) ^2^Pearson's Chi-squared test; Wilcoxon rank sum test ALP, alkaline phosphatase; ALT, alanine aminotransferase; AST, aspartate aminotransferase; BMI, body mass index; BUN, blood urea nitrogen; GGT, gamma-glutamyl transferase; HDL, high-density lipoprotein; LDH, lactate dehydrogenase; MCH, mean corpuscular hemoglobin; MCHC, mean corpuscular hemoglobin concentration; MCV, mean corpuscular volume; MPV, mean platelet volume; RBC, red blood cell count; RDW, red cell distribution width; TB, total bilirubin; WBC, white blood cell count.
