# Supplementary material for: Global trends and risk factors in gastric cancer: a comprehensive analysis of the Global Burden of Disease Study 2021 and multi-omics data
Source: Int J Med Sci. 2025 Jan 1;22(2):341–56. doi: 10.7150/ijms.104437 (PMC11704698; doi:10.7150/ijms.104437)
Supplement: Supplementary file 1 — Supplementary methods and figure. [file ijmsv22p0341s1.pdf]

Supplementary method

Figure 1A delineates the comprehensive, multi-stage research design employed in this investigation of gastric cancer (GC) epidemiology and associated risk factors. The study commenced with an extensive analysis of GC epidemiology utilizing the Global Burden of Disease (GBD) database. This analysis encompassed the examination of age-standardized disability-adjusted life year rates (ASDR) across 204 countries and territories, frontier analysis based on the socio-demographic index (SDI), and the projection of future trends in disability-adjusted life years (DALYs) employing Bayesian Age-Period-Cohort (BAPC) models. Health inequalities were assessed using the slope index of inequality (SII) and concentration index.

Subsequently, the NHANES database was utilized to analyze clinical characteristics associated with GC. Following data cleaning of the NHANES database, we employed propensity score matching (PSM) to extract samples. Subsequently, we conducted analyses using both conventional logistic regression and an array of 16 machine learning models.

Disease and food-liking phenotypes association studies were conducted using the FinnGen R10, UK Biobank SAIGE, *Helicobacter pylori* infection (anti-*Helicobacter pylori* IgG levels), and food-liking databases, incorporating Mendelian Randomization Phenome-Wide Association Study (MR-PheWAS) methodologies to identify phenotypes potentially influencing GC risk.

Further investigations explored the intricate relationships between blood markers, gut microbiota, and GC risk. This phase involved the analysis of various metabolites, immune cell populations, lipid profiles, inflammatory proteins, and microbiota through two-sample Mendelian Randomization (MR) techniques.

The final phase of the study comprised transcriptomic and epigenetic analyses. The Cancer Genome Atlas (TCGA) and Genotype-Tissue Expression (GTEx) databases were employed for differential gene expression analysis, while the eQTLGen dataset were utilized for expression Quantitative Trait Loci (QTL) analysis through Summary-data-based Mendelian Randomization (SMR). The integration of Differentially Expressed Genes (DEGs) and QTLs facilitated the identification of potential protective

and risk genes for GC.

Detailed study design and data resources are as follows.

The design and data resources of GBD analysis

This investigation leveraged data from the Global Burden of Diseases, Injuries, and Risk Factors Study 2021 (GBD 2021), accessible via the Global Health Data Exchange (https://vizhub.healthdata.org/gbd-results). The GBD 2021 represents the most comprehensive epidemiological assessment to date, encompassing 371 diseases and injuries and 88 risk factors across global, regional, and national scales [1]. DALYs were employed as the primary metric of disease burden, amalgamating years of life lost due to premature mortality and years lived with disability. Age-standardized rates (ASRs) were calculated per 100,000 individuals, facilitating comparisons across demographically diverse populations. The SDI, a composite measure ranging from 0 (least developed) to 1 (most developed), integrates income per capita, educational attainment, and fertility rates, enabling temporal and geographical comparisons. The 204 countries and territories under study were stratified into five SDI quintiles: high, high-middle, middle, low-middle, and low.

Our analysis focused on DALYs and DALY rates for individuals aged 20 and above from 1990 to 2021. Estimates are presented as both counts and ASRs per 100,000, accompanied by 95% Uncertainty Intervals (95% UIs). To quantify temporal trends, we calculated the Average Annual Percentage Change (AAPC) in GC ASDR from 1990 to 2021 across genders and SDI quintiles using Joinpoint regression analysis, employing software provided by the US National Cancer Institute Surveillance Research Program [2].

To evaluate trends in health inequality related to GC from 1990 to 2021, we utilized two complementary measures: the SII and the concentration index [3-6]. The SII quantifies absolute inequality, representing the absolute difference in health outcomes between the theoretically most and least advantaged subgroups across the entire socioeconomic spectrum. A positive SII indicates higher ASDR in regions with higher SDI, while a negative SII suggests the converse. The concentration index measures relative inequality, quantifying the degree of socioeconomic-related inequality in health

outcomes. The concentration index ranges from -1 to +1, with 0 representing perfect equality. A negative concentration index indicates a concentration of the health outcome among lower SDI regions, while a positive concentration index suggests concentration among higher SDI regions. Both indices were calculated for 1990 and 2021, enabling comparison of absolute and relative inequalities over the three-decade period.

To elucidate the relationship between sociodemographic progress and GC burden, we conducted a frontier analysis using SDI to identify minimum achievable ASDR [7]. This analysis highlighted countries and territories at the vanguard of GC burden reduction relative to their SDI, with the "effective difference" indicating the gap between current and potentially achievable burdens. Decomposition analyses were performed to understand variations in GC DALYs between 1990 and 2021, considering factors such as age structure, population size, and epidemiologic changes [8]. Finally, we employed BAPC forecasting methodology to project rates and absolute numbers of DALYs, years lived with disability, and years of life lost up to 2030 [9]. This multifaceted analytical approach provides a comprehensive assessment of GC epidemiology, socioeconomic disparities, and future trends, offering valuable insights for health policy and resource allocation.

The design and data resources of clinical characteristics related to the risk of GC

The clinical characteristics analysis pertaining to GC risk is illustrated in Figure 1B. This comprehensive investigation utilized data from the NHANES database, spanning from 2005 to March 2020, encompassing a wide array of demographic, anthropometric, and hematological parameters from both GC patients and non-tumor individuals.

The data preprocessing protocol for the NHANES dataset involved a meticulous, multi-step approach to ensure optimal data quality. Initially, we conducted a thorough assessment of missing values for each clinical characteristic. Variables with 30% or more missing data were identified and subsequently excluded to enhance overall data integrity. We then evaluated the proportion of missing values for each sample, eliminating those with 30% or more absent data points. The resulting dataset comprised clinical characteristics with less than 30% missing values, thus optimizing the dataset for subsequent statistical analyses.

To address the remaining missing values, we employed a sophisticated imputation technique: Multiple Imputation by Chained Equations (MICE). Specifically, we utilized random forest as the imputation algorithm, selected for its capacity to manage complex datasets with heterogeneous variable types and its robustness in capturing non-linear relationships between variables [10]. The imputation process was executed with 100 iterations to ensure convergence on stable imputed values, and a single imputed dataset was generated to maintain analytical simplicity. Post-imputation, we conducted rigorous diagnostic checks to verify the plausibility of the imputed values and to ensure that the imputation process did not introduce unexpected patterns or distortions in the data distribution. This comprehensive approach allowed us to preserve the full sample size while minimizing potential biases associated with missing data, thereby enhancing the robustness and reliability of our subsequent statistical analyses.

The final dataset comprised 31,894 participants (26 GC cases versus 31,868 controls), examining 51 distinct clinical characteristics. These variables encompassed various aspects of renal and liver function, metabolic parameters, electrolyte balance, hematological markers, and anthropometric measurements.

Urinary markers included albumin and creatinine. Liver function was assessed through albumin, ALT (Alanine Aminotransferase), AST (Aspartate Aminotransferase), ALP (Alkaline Phosphatase), GGT (Gamma-Glutamyl Transferase), and TB (Total Bilirubin). Renal function was evaluated using BUN (Blood Urea Nitrogen) and creatinine. Metabolic parameters comprised glucose, glycohemoglobin (HbA1c), cholesterol, HDL (High-Density Lipoprotein), triglycerides, and uric acid. We also measured electrolytes and minerals including calcium, phosphorus, sodium, potassium, chloride, bicarbonate, and iron. Additional biochemical markers included LDH (Lactate Dehydrogenase), total protein, globulin, and osmolality. Anthropometric measurements consisted of weight, standing height, BMI (Body Mass Index), and waist circumference. A complete blood count (CBC) was performed, including WBC (White Blood Cell) count, RBC (Red Blood Cell) count, hemoglobin, hematocrit, MCV (Mean Corpuscular Volume), MCH (Mean Corpuscular Hemoglobin), MCHC (Mean Corpuscular Hemoglobin Concentration), RDW (Red Cell Distribution Width), platelet count, and

MPV (Mean Platelet Volume). The differential white blood cell count included percentages and absolute counts (where applicable) of lymphocytes, monocytes, neutrophils, eosinophils, and basophils.

Given the highly skewed distribution of our dataset (26 cases versus 31,868 controls), which could potentially introduce significant bias, we employed propensity score matching (PSM). This method matched cases to controls at a 1:10 ratio, based on gender and age, to mitigate potential confounding effects and enhance the validity of our analysis [11]. The matching process yielded a final sample of 286 subjects (26 cases vs. 260 controls). The efficacy of PSM was evaluated using several statistical measures. Standardized Mean Difference (SMD): Assessed the covariate distribution difference between GC and healthy groups, with lower values indicating more effective matching. Variance ratio: Compared the variance of covariates between groups, with ratios between 0.5 and 2 considered acceptable, indicating satisfactory matching quality. Empirical cumulative distribution function (eCDF) mean and max: Evaluated the cumulative distribution functions of covariates, with lower values indicating a closer match in covariate distribution. To visually represent these distributions, we utilized density plot and bar graph, providing a graphical representation of the matching quality. These combined statistical and visual methods offered a comprehensive assessment of the PSM's efficacy in balancing covariate distribution between the GC and healthy groups.

For intergroup comparisons of clinical characteristics, we employed Pearson's Chi-squared test for categorical variables and the Wilcoxon rank-sum test for continuous variables. Multivariate logistic regression analysis was conducted to explore associations between 49 clinical characteristics (excluding age and gender) and GC risk, with statistical significance set at $p<0.05$.

Despite the application of PSM, a slight imbalance persisted in the dataset. Recognizing the potential limitations of conventional logistic regression under these circumstances, we implemented a more comprehensive approach. We utilized an ensemble of 16 machine learning models in conjunction with SHAP (SHapley Additive exPlanations) interpreters to elucidate the associations between clinical characteristics and GC risk.

This approach facilitated robust feature selection and model optimization, potentially uncovering nuanced relationships that might be overlooked by traditional statistical methods.

The machine learning models employed encompassed a diverse range of algorithms: Artificial Neural Network (ANN), AdaBoost, Naive Bayes, CatBoost, Decision Tree, Elastic Net, Gradient Boosting, K-Nearest Neighbors (KNN), Light Gradient Boosting Machine (LGBM), Lasso Regression, Linear Discriminant Analysis (LDA), Quadratic Discriminant Analysis (QDA), Random Forest, Ridge Regression, Support Vector Machine (SVM), and XGBoost.

Prior to machine learning modeling, we standardized the clinical characteristics data (excluding age and gender) using z-score normalization [12, 13]. This process transformed each feature to have a mean of 0 and a standard deviation of 1, facilitating the comparison and analysis of variables with originally disparate scales and units. The standardized dataset was then partitioned into training and test sets at a 7:3 ratio.

To address the residual class imbalance, we applied the Adaptive Synthetic (ADASYN) sampling technique to the training set. ADASYN, an advanced oversampling method, generates synthetic samples for the minority class (GC cases) adaptively based on their learning difficulty. This approach not only balances the dataset but also shifts the classification decision boundary towards more challenging examples, potentially enhancing the model's discriminative capacity between GC cases and controls [14]. For each of the 16 machine learning models, we employed the area under the receiver operating characteristic curve (AUC) in the test set to guide feature selection. The preliminary models were then fine-tuned using grid search with predefined parameter grids. Finally, we aggregated and visualized the top 3 parameter combinations yielding the highest AUC values in the test set for each machine learning model, providing a comprehensive overview of model performance and optimization.

To elucidate the complex relationships between clinical characteristics and GC risk identified by our machine learning models, we employed SHAP [15]. SHAP, a game theory-based approach, assigns each feature an importance value for a particular prediction, providing a unified measure of feature importance that is consistent, locally

accurate, and capable of handling feature interactions.

Following the optimization of our 16 machine learning models, we applied SHAP to each model utilizing the AUC in the test set. We then generated a comprehensive summary plot of SHAP values for each variable across all models, offering a holistic view of feature importance. To further elucidate the relative significance of each variable, we assessed the ranking of SHAP values within individual models and the frequency of each variable's appearance across the ensemble of 16 models. These results were visualized using bar plots, providing an intuitive understanding of variable importance.

To elucidate the specific performance of each machine learning model in predicting GC, we employed waterfall plots and decision plots for the test set. These visualizations offered detailed insights into how individual variables influenced the model's decision-making process in classifying samples as either GC or control. Additionally, we utilized beeswarm plots on the test set to visualize the relationship between various clinical characteristics and GC risk. This approach allowed for clear observation of how dynamic changes in the values of each clinical characteristic corresponded to changes in GC risk. This comprehensive analysis using SHAP not only enhanced the interpretability of our complex machine learning models but also provided clinically relevant insights into the factors associated with GC risk.

The design and data resources of MR analysis

We employed MR-PheWAS analyses to elucidate potential causal relationships between various disease phenotypes, food preferences, blood markers, and microbiota composition with GC risk. Our two-sample MR framework was predicated on three fundamental assumptions (Figure 1C): (1) robust association between genetic instrumental variables (IVs) and exposure factors, (2) independence from confounding variables, and (3) influence on outcomes exclusively through exposure factors [16, 17]. The MR analysis followed a rigorous, systematic approach to examine putative causal relationships between exposures and outcomes (Figure 1D). We initiated our investigation by extracting data from genome-wide association studies (GWAS). Eligible IVs were selected based on their significant association with exposure variables,

employing variable thresholds for p-values across different exposure variables (detailed in Supplementary Table S14). We subsequently filtered IVs associated with the outcome using a threshold of 5×10^{-5}. To refine our selection further, we implemented the Steiger test method, which evaluates the relative strength of each single nucleotide polymorphism's (SNP) association with the exposure versus the outcome [18]. This crucial step ensures that selected IVs primarily influence the exposure rather than directly affecting the outcome, thereby enhancing the validity of our MR analysis. To mitigate potential bias from linkage disequilibrium (LD), we removed SNPs in LD using variable thresholds for r^2 (ranging from 0.001 to 0.1) across distances spanning 500 kb to 10,000 kb, tailored to each specific dataset [19]. We assessed the strength of each IV using F-statistics to address potential weak IV bias, considering an F statistic below 10 indicative of a weak IV [20, 21]. In our study, the reported F-statistics represent the mean of the aggregated F-statistics for individual SNPs, calculated using the formula: $(beta/SE)^2$, where beta denotes the estimate of the IV's effect size, and SE represents the standard error of this estimate [22]. Weak IVs were subsequently excluded from further analysis. After obtaining a set of independent, non-LD IVs, we extracted the corresponding SNPs for the outcome variable. We then applied the harmonize effect method to align effect sizes and alleles across exposure and outcome datasets, resulting in a refined set of effective IVs for our analysis. Utilizing these harmonized data, we conducted two-sample MR analyses to estimate the causal effect of exposures on outcomes.

Statistical analyses were performed using the "Two Sample MR," "Mendelian Randomization," and "RadialMR" packages in R. The inverse-variance weighted (IVW) method, implemented under a random-effects model, served as our primary approach for estimating potential bidirectional causal relationships between exposure and outcome variables. This method assumes strict adherence to fundamental MR principles. We also employed MR-Egger, weighted median, and weighted mode methods as complementary approaches, with results provided for reference.

To ensure robust causal inference, we conducted comprehensive sensitivity analyses [23, 24]. These analyses included assessments of heterogeneity and pleiotropy, with a

p-value below 0.05 considered statistically significant. Heterogeneity was evaluated using both the IVW and MR-Egger methods, interpreted via Cochran's Q-test P-value [25]. Horizontal pleiotropy was assessed using the Egger intercept method [26]. Our MR analysis conclusions were primarily based on findings derived from the IVW method, with the caveat that results should not exhibit heterogeneity or pleiotropy to be considered valid. This rigorous methodological approach enhances the reliability and interpretability of our findings, providing valuable insights into the complex etiology of GC.

This study utilized a comprehensive array of datasets to conduct robust MR analyses, with detailed information presented in Supplementary Table S14. The primary outcome dataset for GC was derived from the GWAS catalog, encompassing a diverse cohort of 1,029 European ancestry cases and 475,087 controls, alongside 7,921 East Asian ancestry cases and 159,201 controls [27]. For exposure data, we leveraged several large-scale resources. The FinnGen R10 dataset provided phenotypic information on 2,408 traits across 412,181 individuals. The UK Biobank SAIGE dataset contributed data on 783 phenotypes, with case numbers ranging from 51 to 77,977 and control numbers from 330,366 to 408,908. There are 4,683 samples in the data set of *Helicobacter pylori* infection (anti-*Helicobacter pylori* IgG levels) [28]. Food preferences were assessed using a 9-point scale for 139 specific foods in a cohort of 161,625 individuals [29]. We also augmented our analysis with specialized datasets, including 1,400 blood metabolites and metabolite ratios (n = 8,299), 731 immune cell traits (n = 3,757), 179 lipid species (n = 7,174), and 91 inflammatory proteins (n = 14,824) [30-33]. Gut microbiota data were sourced from two distinct cohorts: a Finnish cohort providing data on 471 distinct Genome Taxonomy Database taxa (n = 5,959), and a German cohort offering abundance and prevalence data for 430 microbiome traits (n = 8,956) [34, 35]. Our stringent methodological approach involved filtering SNPs associated with the outcome using a threshold of 5×10^{-5} across all datasets. For phenotypic data, IV selection generally employed a threshold of 5×10^{-6} for exposures, with LD analysis performed using an r^2 threshold of 0.001 within distances of 10,000 kb. For other datasets, IV selection utilized a threshold of 1×10^{-5} for exposures, with

LD analysis conducted using an r^2 threshold of 0.1 within distances of 500 kb.

This meticulous data curation and analysis strategy ensures a comprehensive exploration of potential causal factors influencing GC risk, spanning a wide array of phenotypes, metabolites, immune markers, lipid species, inflammatory proteins, and microbial taxa. The incorporation of diverse populations and large sample sizes enhances the generalizability and statistical power of our findings, providing a solid foundation for identifying novel risk factors and potential therapeutic targets in GC etiology.

The design and data resources of genes analysis

In this comprehensive investigation, we elucidated the intricate relationship between genetic factors and GC risk. Candidate genes were stratified into three evidence levels, with level 1 representing the strongest and level 3 the weakest empirical support. We conducted an extensive bioinformatics analysis utilizing data from TCGA and GTEx databases [36, 37]. The initial cohort comprised 623 samples (413 GC cases and 210 controls), with gene expression quantified as raw count data. Following stringent quality control measures, including the exclusion of genes expressed in less than 50% of samples and logarithmic transformation of counts per million (CPM) normalized data, we performed Principal Component Analysis (PCA) for sample assessment (Figure 1E). This rigorous process led to the identification and subsequent removal of two outlier control samples, yielding a final dataset of 621 samples (413 cases and 208 controls) (Figure 1F).

Differential gene expression analysis was executed using the "DESeq2" R package, a robust tool optimized for count-based RNA-sequencing data [38]. Our analytical pipeline encompassed normalization, dispersion estimation, and statistical inference via the Wald test. Employing a significance threshold of $p<0.05$, we identified 15,369 DEGs between GC tissues and healthy controls (evidence level 3). To elucidate the expression patterns of these DEGs, we generated a heatmap depicting the top 1000 DEGs across all samples, providing a clear visual representation of gene expression disparities between GC tissues and control groups.

To investigate the potential mediation of SNP effects on GC risk through gene

expression, we employed SMR multi-tools [39, 40]. We leveraged blood expression quantitative trait loci (eQTL) summary statistics from the eQTLGen consortium, encompassing 31,684 individuals across 37 datasets, with 16,987 genes tested for cis-eQTLs and 6,298 for trans-eQTLs [41]. Our analysis focused exclusively on cis-regulatory elements, defined as SNPs within a 2-Mb window of the gene's transcriptional start and end sites. A two-step SMR analysis was conducted: first utilizing SNPs as instruments, blood gene expressions as exposure, and GC as the outcome. Candidate genes were identified based on meeting both SMR criteria ($p < 0.05$) and demonstrating suggestive genome-wide significance ($p < 1 \times 10^{-5}$) in all eQTLs. We implemented a Heterogeneity in Dependent Instruments (HEIDI) test, with a threshold of $p < 0.05$, to identify and characterize this heterogeneity. Heterogeneity is not allowed in the result of this study.

To synthesize our findings, we performed an intersection analysis of the DEGs identified through bioinformatics analysis and genes highlighted by SMR analysis. This integration was visually represented through a Venn diagram. This rigorous selection process yielded our set of candidate genes (evidence level 2). To visually represent our results, we developed a circular heatmap to provide a holistic view of gene expression patterns in GC compared to controls. These visualizations effectively demonstrate the differential expression of our candidate genes between GC and healthy tissues, as well as the intricate relationships among their expression levels and GC risk.

To further refine our selection of robust candidate genes, we implemented a sophisticated colocalization analysis [42, 43]. This advanced statistical approach was employed to identify overlapping genetic variants potentially responsible for distinct traits, thereby enhancing our capacity to detect putative causal single nucleotide polymorphisms (SNPs). Specifically, we integrated genome-wide association study GWAS data with QTL data, facilitating a more nuanced understanding of the genetic architecture underlying GC susceptibility. This method evaluates the presence of a shared causal variant in a given genomic region for two traits of interest, namely gene expression and GC susceptibility. We utilized the "coloc" R package, a well-established tool in genetic epidemiology, to conduct this analysis [44, 45]. The package implements

a Bayesian statistical framework to estimate the probability of colocalization between two genetic association signals. In our analysis, we applied stringent criteria for determining shared genetic effects between the two traits. Specifically, we employed a posterior probability of hypothesis 4 (PP.H4) greater than 0.5 as our threshold [46, 47]. The PP.H4 represents the probability that both traits are associated with the same causal variant in the region. This threshold ensures a high level of confidence in our identified colocalized signals, minimizing false positives while maintaining sensitivity to detect true shared genetic effects. Through this rigorous colocalization analysis, we identified candidate genes with the highest level of evidence (level 1). To visually represent the results of our colocalization analysis, we generated multiple graphical depictions. A volcano plot was constructed to illustrate the significance and magnitude of gene expression changes. An MA plot was created to delineate the relationship between mean expression levels and log-fold changes. We also employed the R package 'locuscomparer' to generate colocalization plots [48]. In these plots, the lead SNP is prominently labeled, while other SNPs are color-coded based on their LD with the lead SNP, providing a visual representation of the genetic landscape associated with GC risk. All statistical analyses and data visualizations in this study were performed using R version 4.3.1 (R Core Team, 2023) and Python version 3.9 (Python Software Foundation, https://www.python.org/).

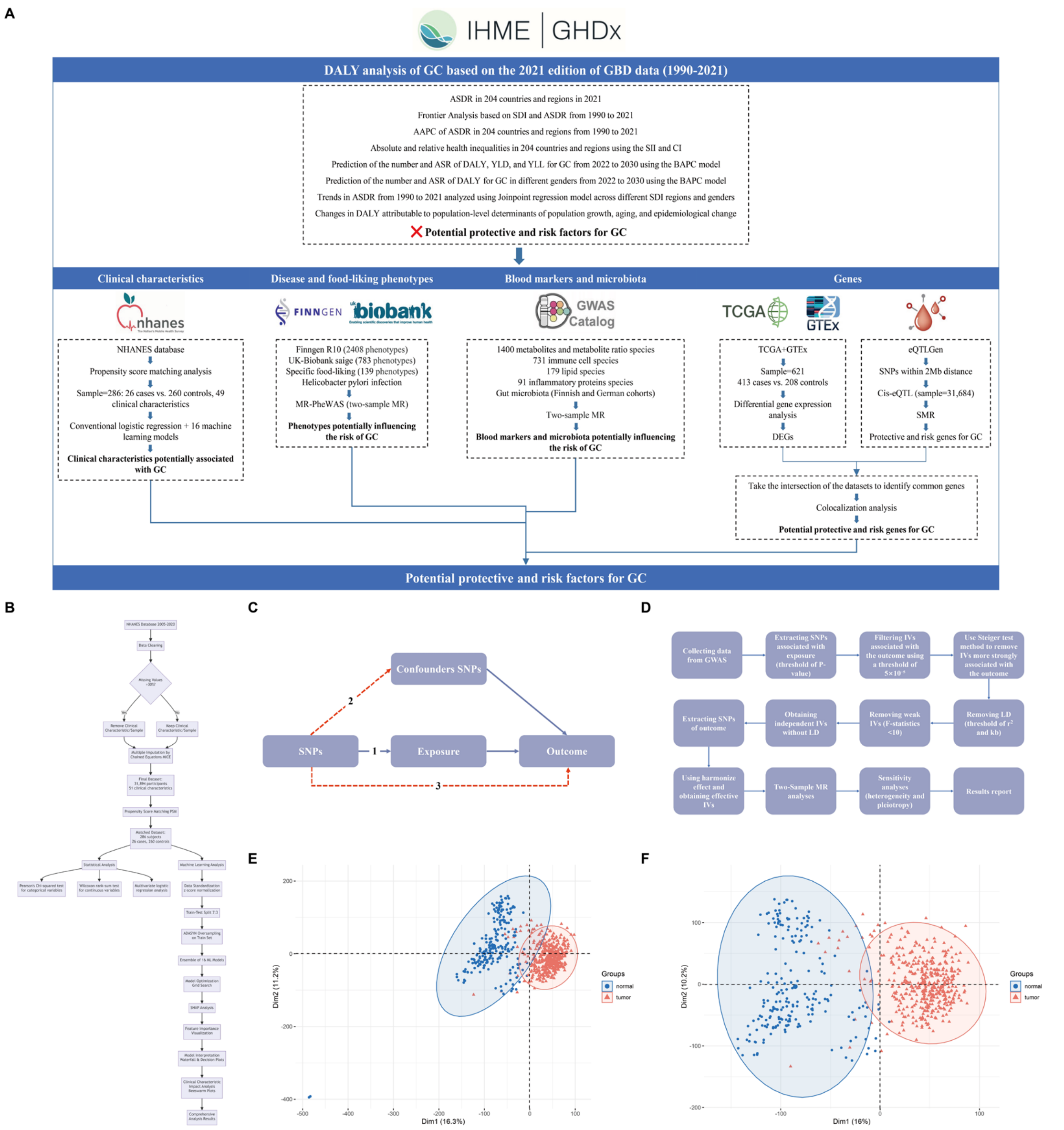

Figure 1. Schematic overview of the study design and methodologies. (A) Flowchart of the overall research process. (B) Flowchart depicting the analysis of clinical characteristics associated with gastric cancer (GC) risk. (C) The three critical assumptions underlying Mendelian Randomization (MR) analysis. (D) Comprehensive workflow of the Mendelian Randomization analysis. (E) Principal Component Analysis (PCA) plot before the removal of outlier samples. (F) Principal Component Analysis (PCA) plot after the removal of outlier samples.

References

1. Burden of disease scenarios for 204 countries and territories, 2022-2050: a forecasting analysis for the Global Burden of Disease Study 2021. Lancet. 2024; 403: 2204-56.
2. Kim HJ, Fay MP, Feuer EJ, Midthune DN. Permutation tests for joinpoint regression with applications to cancer rates. Stat Med. 2000; 19: 335-51.
3. Wagstaff A, Paci P, van Doorslaer E. On the measurement of inequalities in health. Soc Sci Med. 1991; 33: 545-57.
4. Mackenbach JP, Kunst AE. Measuring the magnitude of socio-economic inequalities in health:

an overview of available measures illustrated with two examples from Europe. Soc Sci Med. 1997; 44: 757-71.
5. Regidor E. Measures of health inequalities: part 2. J Epidemiol Community Health. 2004; 58: 900-3.
6. Moreno-Betancur M, Latouche A, Menvielle G, Kunst AE, Rey G. Relative index of inequality and slope index of inequality: a structured regression framework for estimation. Epidemiology. 2015; 26: 518-27.
7. Xie Y, Bowe B, Mokdad AH, Xian H, Yan Y, Li T, et al. Analysis of the Global Burden of Disease study highlights the global, regional, and national trends of chronic kidney disease epidemiology from 1990 to 2016. Kidney Int. 2018; 94: 567-81.
8. Gupta PD. Standardization and decomposition of rates: A user's manual: US Department of Commerce, Economics and Statistics Administration, Bureau …; 1993.
9. Riebler A, Held LJBJ. Projecting the future burden of cancer: Bayesian age–period–cohort analysis with integrated nested Laplace approximations. 2017; 59: 531-49.
10. Van Buuren S, Groothuis-Oudshoorn K. mice: Multivariate imputation by chained equations in R. Journal of statistical software. 2011; 45: 1-67.
11. Austin PC. Balance diagnostics for comparing the distribution of baseline covariates between treatment groups in propensity-score matched samples. Stat Med. 2009; 28: 3083-107.
12. Jain A, Zongker D. Feature selection: Evaluation, application, and small sample performance. IEEE transactions on pattern analysis and machine intelligence. 1997; 19: 153-8.
13. Aksoy S, Haralick RM. Feature normalization and likelihood-based similarity measures for image retrieval. Pattern recognition letters. 2001; 22: 563-82.
14. He H, Bai Y, Garcia EA, Li S. ADASYN: Adaptive synthetic sampling approach for imbalanced learning. 2008 IEEE international joint conference on neural networks (IEEE world congress on computational intelligence): Ieee; 2008. p. 1322-8.
15. Lundberg SM, Lee S-I. A unified approach to interpreting model predictions. Advances in neural information processing systems. 2017; 30.
16. Davey Smith G, Hemani G. Mendelian randomization: genetic anchors for causal inference in epidemiological studies. Hum Mol Genet. 2014; 23: R89-98.
17. Holmes MV, Ala-Korpela M, Smith GD. Mendelian randomization in cardiometabolic disease: challenges in evaluating causality. Nat Rev Cardiol. 2017; 14: 577-90.
18. Hemani G, Tilling K, Davey Smith G. Orienting the causal relationship between imprecisely measured traits using GWAS summary data. PLoS Genet. 2017; 13: e1007081.
19. Chang CC, Chow CC, Tellier LC, Vattikuti S, Purcell SM, Lee JJ. Second-generation PLINK: rising to the challenge of larger and richer datasets. Gigascience. 2015; 4: 7.
20. Pierce BL, Ahsan H, Vanderweele TJ. Power and instrument strength requirements for Mendelian randomization studies using multiple genetic variants. Int J Epidemiol. 2011; 40: 740-52.
21. Burgess S, Thompson SG. Avoiding bias from weak instruments in Mendelian randomization studies. Int J Epidemiol. 2011; 40: 755-64.
22. Huang W, Xiao J, Ji J, Chen L. Association of lipid-lowering drugs with COVID-19 outcomes from a Mendelian randomization study. Elife. 2021; 10.
23. Bowden J, Davey Smith G, Burgess S. Mendelian randomization with invalid instruments: effect estimation and bias detection through Egger regression. Int J Epidemiol. 2015; 44: 512-25.

24. Bowden J, Davey Smith G, Haycock PC, Burgess S. Consistent Estimation in Mendelian Randomization with Some Invalid Instruments Using a Weighted Median Estimator. Genet Epidemiol. 2016; 40: 304-14.
25. Bowden J, Spiller W, Del Greco MF, Sheehan N, Thompson J, Minelli C, et al. Improving the visualization, interpretation and analysis of two-sample summary data Mendelian randomization via the Radial plot and Radial regression. Int J Epidemiol. 2018; 47: 1264-78.
26. Bowden J, Holmes MV. Meta-analysis and Mendelian randomization: A review. Res Synth Methods. 2019; 10: 486-96.
27. Sakaue S, Kanai M, Tanigawa Y, Karjalainen J, Kurki M, Koshiba S, et al. A cross-population atlas of genetic associations for 220 human phenotypes. Nat Genet. 2021; 53: 1415-24.
28. Chong AHW, Mitchell RE, Hemani G, Davey Smith G, Yolken RH, Richmond RC, et al. Genetic Analyses of Common Infections in the Avon Longitudinal Study of Parents and Children Cohort. Front Immunol. 2021; 12: 727457.
29. May-Wilson S, Matoba N, Wade KH, Hottenga JJ, Concas MP, Mangino M, et al. Large-scale GWAS of food liking reveals genetic determinants and genetic correlations with distinct neurophysiological traits. Nat Commun. 2022; 13: 2743.
30. Chen Y, Lu T, Pettersson-Kymmer U, Stewart ID, Butler-Laporte G, Nakanishi T, et al. Genomic atlas of the plasma metabolome prioritizes metabolites implicated in human diseases. Nat Genet. 2023; 55: 44-53.
31. Orrù V, Steri M, Sidore C, Marongiu M, Serra V, Olla S, et al. Complex genetic signatures in immune cells underlie autoimmunity and inform therapy. Nat Genet. 2020; 52: 1036-45.
32. Ottensmann L, Tabassum R, Ruotsalainen SE, Gerl MJ, Klose C, Widén E, et al. Genome-wide association analysis of plasma lipidome identifies 495 genetic associations. Nat Commun. 2023; 14: 6934.
33. Zhao JH, Stacey D, Eriksson N, Macdonald-Dunlop E, Hedman Å K, Kalnapenkis A, et al. Genetics of circulating inflammatory proteins identifies drivers of immune-mediated disease risk and therapeutic targets. Nat Immunol. 2023; 24: 1540-51.
34. Qin Y, Havulinna AS, Liu Y, Jousilahti P, Ritchie SC, Tokolyi A, et al. Combined effects of host genetics and diet on human gut microbiota and incident disease in a single population cohort. Nat Genet. 2022; 54: 134-42.
35. Rühlemann MC, Hermes BM, Bang C, Doms S, Moitinho-Silva L, Thingholm LB, et al. Genome-wide association study in 8,956 German individuals identifies influence of ABO histo-blood groups on gut microbiome. Nat Genet. 2021; 53: 147-55.
36. Comprehensive molecular characterization of gastric adenocarcinoma. Nature. 2014; 513: 202-9.
37. The Genotype-Tissue Expression (GTEx) project. Nat Genet. 2013; 45: 580-5.
38. Love MI, Huber W, Anders S. Moderated estimation of fold change and dispersion for RNA-seq data with DESeq2. Genome Biol. 2014; 15: 550.
39. Zhu Z, Zhang F, Hu H, Bakshi A, Robinson MR, Powell JE, et al. Integration of summary data from GWAS and eQTL studies predicts complex trait gene targets. Nat Genet. 2016; 48: 481-7.
40. Qi T, Wu Y, Zeng J, Zhang F, Xue A, Jiang L, et al. Identifying gene targets for brain-related traits using transcriptomic and methylomic data from blood. Nature Communications. 2018; 9: 2282.
41. Võsa U, Claringbould A, Westra HJ, Bonder MJ, Deelen P, Zeng B, et al. Large-scale cis- and

trans-eQTL analyses identify thousands of genetic loci and polygenic scores that regulate blood gene expression. Nat Genet. 2021; 53: 1300-10.
42. Wallace C. Eliciting priors and relaxing the single causal variant assumption in colocalisation analyses. PLoS Genet. 2020; 16: e1008720.
43. Giambartolomei C, Vukcevic D, Schadt EE, Franke L, Hingorani AD, Wallace C, et al. Bayesian test for colocalisation between pairs of genetic association studies using summary statistics. PLoS Genet. 2014; 10: e1004383.
44. Wang G, Sarkar A, Carbonetto P, Stephens M. A simple new approach to variable selection in regression, with application to genetic fine mapping. J R Stat Soc Series B Stat Methodol. 2020; 82: 1273-300.
45. Wallace C. A more accurate method for colocalisation analysis allowing for multiple causal variants. PLoS Genet. 2021; 17: e1009440.
46. Huang Y, Shan Y, Zhang W, Lee AM, Li F, Stranger BE, et al. Deciphering genetic causes for sex differences in human health through drug metabolism and transporter genes. Nat Commun. 2023; 14: 175.
47. Arvanitis M, Tayeb K, Strober BJ, Battle A. Redefining tissue specificity of genetic regulation of gene expression in the presence of allelic heterogeneity. Am J Hum Genet. 2022; 109: 223-39.
48. Liu B, Gloudemans MJ, Rao AS, Ingelsson E, Montgomery SB. Abundant associations with gene expression complicate GWAS follow-up. Nature Genetics. 2019; 51: 768-9.
